# Metabolomic Profiles Reveal Potential Factors that Correlate with Lactation Performance in Sow Milk

**DOI:** 10.1038/s41598-018-28793-0

**Published:** 2018-07-16

**Authors:** Chengquan Tan, Zhenya Zhai, Xiaojun Ni, Hao Wang, Yongcheng Ji, Tianyue Tang, Wenkai Ren, Hongrong Long, Baichuan Deng, Jinping Deng, Yulong Yin

**Affiliations:** 1 Guangdong Provincial Key Laboratory of Animal Nutrition Control, Institute of Subtropical Animal Nutrition and Feed, College of Animal Science, South China Agricultural University, Guangzhou, 510642 P.R. China; 2 Jiangsu Co-Innovation Center for Important Animal Infectious Diseases and Zoonoses, Joint International Research Laboratory of Agriculture and Agri-Product Safety of Ministry of Education of China, College of Veterinary Medicine, Yangzhou University, Yangzhou, 225009 China; 3 National Engineering Laboratory for Pollution Control and Waste Utilization in Livestock and Poultry Production, Institute of Subtropical Agriculture, The Chinese Academy of Sciences, Changsha, 410125 P.R. China

## Abstract

Sow milk contains necessary nutrients for piglets; however, the relationship between the levels of metabolites in sow milk and lactation performance has not been thoroughly elucidated to date. In this study, we analysed the metabolites in sow milk from Yorkshire sows with high lactation (HL) or low lactation (LL) performance; these categories were assigned based on the weight gain of piglets during the entire lactation period (D1 to D21). The concentration of milk fat in the colostrum tended to be higher in the HL group (P = 0.05), the level of mannitol was significantly lower in the HL group (*P* < 0.05) and the level of glucuronic acid lactone was significantly higher in the HL group (*P* < 0.05) compared to those in LL group. In mature milk, the levels of lactose, creatine, glutamine, glutamate, 4-hydroxyproline, alanine, asparagine, and glycine were significantly higher (*P* < 0.05) in the HL group than those in LL group. The level of fatty acids showed no significant difference between the two groups in both the colostrum and mature milk. This study suggested that lactation performance may be associated with the levels of lactose and several amino acids in sow milk, and these results can be used to develop new feed additives to improve lactation performance in sows.

## Introduction

Lactation performance in sows is calculated based on weight gain of piglets throughout the entire lactation period (D1 to D21 for weaning)^1^. To date, the studies on improving lactation performance of sows have focused on dietary supplements or adjustment of feed formula. For instance, dietary supplementation with high-calorie materials (e.g., tallow, corn oil and glycerol) increases the lactation performance of sows^2–5^. Additionally, amino acids (e.g., lysine, methionine) and metallic elements (e.g., copper) increase the lactation performance of sows^6^. Notably, previous studies showed that the metabolic status of sows might be the key factor to affect their lactation performance^7^. Indeed, the drastic catabolism and anabolism during lactation may induce pathologies in sows, and oxidative stress, obesity, insulin resistance and inflammation are common phenomena in pregnant or postpartum sows, which prevent sows from secreting higher quality milk^8,9^.

Milk is a complex biological fluid produced by the mammary glands of mammals and is considered to be the gold standard for researchers to understand nutritional requirements of infants^10^. Currently, the components of cow milk and human breast milk are widely studied because they are used as food sources for human infants. For example, metabolites in milk can reflect the metabolic status of mammary glands and the peripheral blood^11^. More than 700 small molecular metabolites have been detected in human milk^12^, and all of them can be classified as carbohydrates, lipids, amino acids, vitamins, and corresponding derivates. Various factors, including species, diet, lactation stage and environment, affect the milk composition^13–15^. Compared to human milk and cow milk, sow milk contains higher levels of fat (6–9%), protein (5–6%), and lactose (4–7%)^16–18^. Additionally, milk also contains various small molecular metabolites (e.g., carbohydrates, amino acids, vitamins and nucleotides), which are also important to the growth and health of infants. For example, glutamate and oligosaccharides not only can be metabolized to provide energy, but are also essential factors for intestinal barrier development, microflora regulation, and immune cell activation^19^. Such vitamins as choline are essential for the nervous system and muscle development for infants^20,21^. As the required materials for RNA synthesis, nucleotides may also regulate the immunity of intestinal cells^20,21^ and improve intestinal blood flow^22^. Thus, the metabolites in sow milk may significantly influence the lactation performance of sows, and identification and integrative analysis of metabolites can provide comprehensive understanding of the relationship between sow milk and lactation performance. Metabolomics provides comprehensive and quantitative analysis of metabolites and aims to establish relationships between genotypes and phenotypes using such tools as mass spectrometry (MS) and nuclear magnetic resonance (NMR)^23–25^. Previous metabolomic studies primarily investigated the breast milk or milk from other mammals, such as cows and donkeys^26,27^. To the best of our knowledge, few metabolomics studies have attempted to identify the metabolites in sow milk^25^.

In this study, we applied metabolomic profiling to characterize the small-molecule metabolites in sow milk with varying lactation performance, primarily focusing on amino acid, carbohydrate, and lipid metabolism pathways, by utilizing gas chromatography-mass spectrometry (GC-MS) and liquid chromatography-mass spectrometry (LC-MS). The results of this study can help to elucidate the difference in composition between high- and low-quality milk. These findings may be beneficial in the development of new feed additives to improve the lactation performance of sows.

## Results

In this study, the components of sow milk were systematically explored through detection of the components present in large quantities (e.g., protein, fat, lactose) and their metabolites. The concentration of protein and lactose in colostrum showed no significant difference (*P* > 0.05) between the HL and LL groups, while the level of fat in HL group tended to be higher (*P* = 0.06) than in LL group (Table 1). In mature milk, the level of fat and protein did not differ significantly between the two groups (*P* > 0.05), while the level of lactose in the HL group was significantly higher than in LL group (*P* < 0.05).

**Table 1:** Concentrations of fat, protein, lactose, and total solid in colostrum and mature milk of sows.

	Group	No. of sows	Fat (%)	Protein (%)	Lactose (%)	Total solid (%)
Colostrum	LL	15	4.65	19.06	4.26	34.92
	HL	15	5.66	18.98	4.30	35.71
	*SEM*		0.50	1.21	0.20	1.29
	*P*-Value		0.05	0.95	0.84	0.55
Milk	LL	15	8.24	6.14	6.80	28.16
	HL	15	7.59	5.94	7.13	28.90
	*SEM*		0.40	0.16	0.10	0.44
	*P*-Value		0.12	0.23	<0.05	0.10

*SEM*: Standard Error of Mean, n = 15 for each group, LL: low lactation performance group, HL: high lactation performance group.

### Identification and quantification of metabolites in colostrum and milk

The representative metabolites involved in carbohydrate, lipid and amino acid metabolism examined in this study are shown in Table 2 and all the metabolites we detected were shown in supplementary information. We primarily targeted amino acids and their derivates, carbohydrates, and lipids. Collectively, 116 metabolites were detected in colostrum and milk by GC-MS and LC-MS. The species of metabolites that could be detected by GC-MS or LC-MS differed. Glycine, tyrosine, glutamate, creatinine and other 15 metabolites can be detected by both platforms, while asparagine, histamine and other 36 metabolites were only detected by LC-MS, and galacturonic acid and other 61 metabolites were only detected by GC-MS.

**Table 2:** Metabolites and metabolic pathways related to lactation performance of sows.

	Metabolic pathway	Metabolites	Method
Carbohydrate metabolism	Glucolysis metabolism, alactose metabolism	Lactic acid, Fucose, Sorbose, glucose, galactose, allose, Ribose, Glucose 6-phosphate, Galacturonic acid, Mannitol, Rhamnose, Ribulose, Glucuronic acid lactone	GC-MS
Amino acid metabolism	Arginine and proline metabolism, Amino acid synthesis, Alanine, aspartate and glutamate metabolism, Glycine, serine and threonine metabolism	Cystine, Asparagine, Creatinine, Aspartic acid, Serine, Leucine, Isoleucine, Valine, Tryptophan, Citrulline, Cysteine, Proline, Creatine, Phenylalanine, Glutamine, Glutamate, Methionine, Arginine	GC-MS/LC-MS
Lipid metabolism	Glycerolipid metabolism, Fatty acid degradation	Glycerol 3-phosphate, Glycerol, Stearic acid, Cholesterol, Oleic acid, Linoleic acid, Palmitoleic	GC-MS

**Figure 1:**
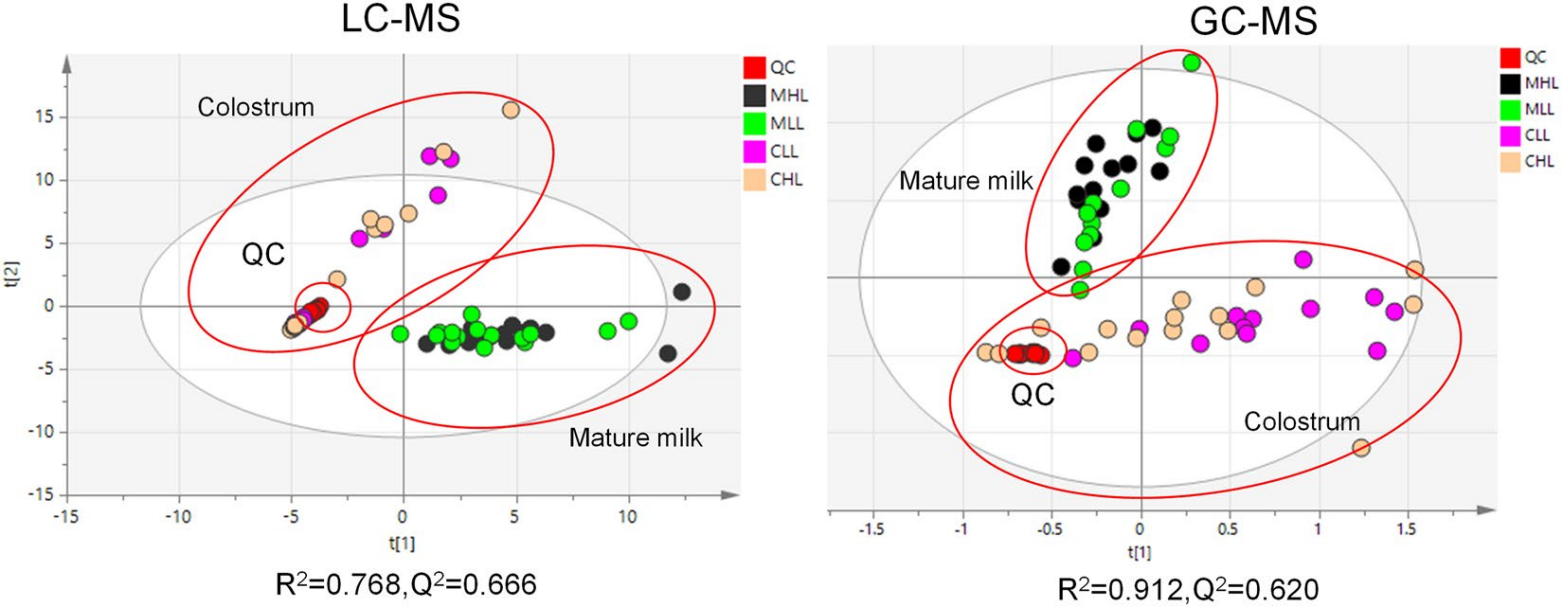
Score plot of PCA model built on data obtained from colostrum and mature milk by using LC-MS (**A**) or GC-MS (**B**) platforms. QC: quality control samples; MHL: mature milk samples in HL group; MLL: mature milk samples in LL group; CHL: colostrum samples in HL group; CLL: colostrum samples in LL group.

In this study, the differences between colostrum and mature milk were analysed. The PCA analyses of LC-MS or GC-MS data were performed separately. To ensure the reliability of the data, quality control (QC) samples were used and demonstrated good reproducibility (Fig. 1). The PCA score plot showed clear separation between colostrum and mature milk (Fig. 1). These results suggest that the metabolite difference between HL and LL groups should be analysed separately for two lactation stages.

### Analysis of differential metabolites

**Figure 2:**
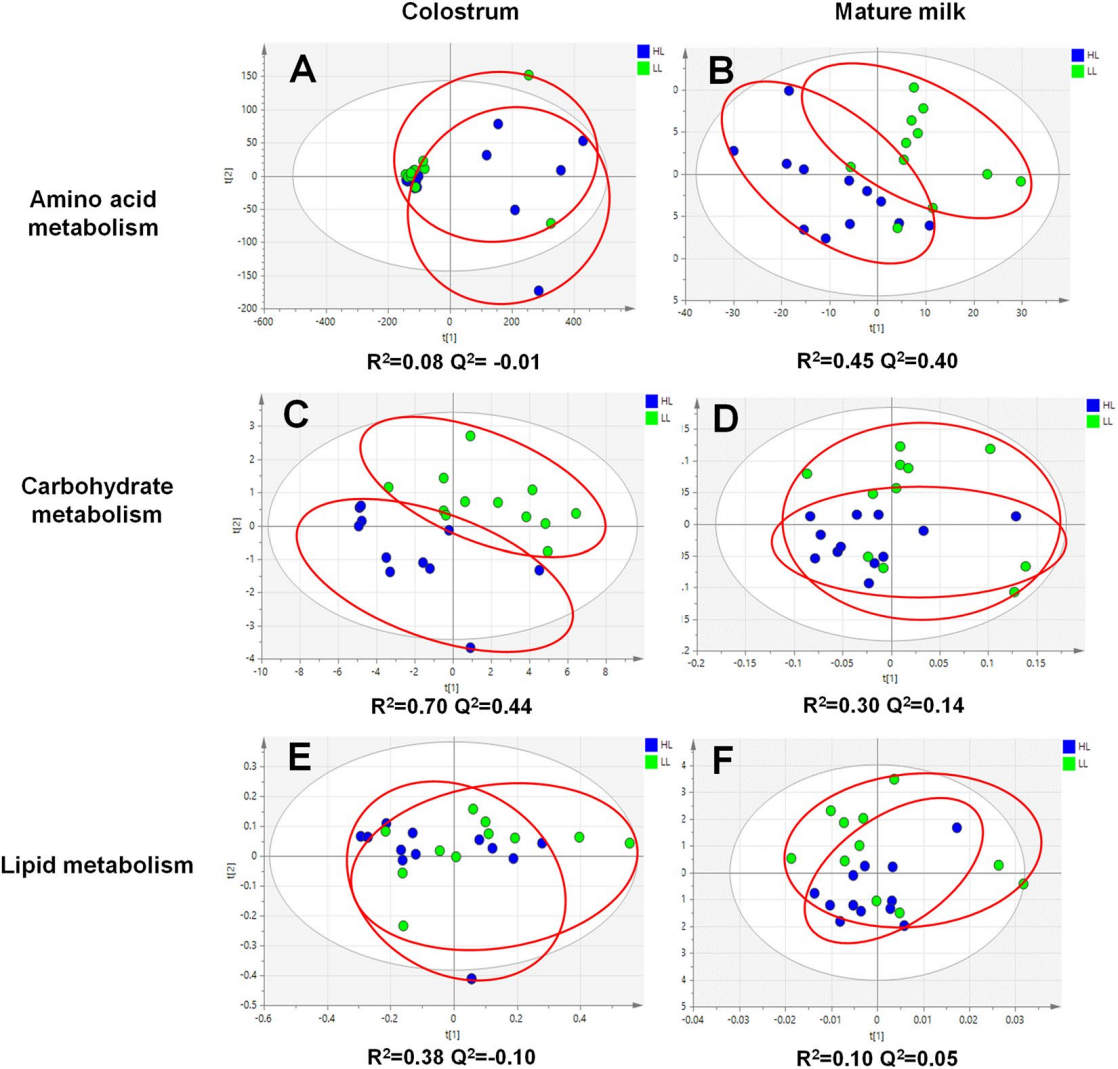
Score plot of PLS-DA model of amino acid, carbohydrate, and lipid metabolism in colostrum and mature milk. HL and LL group are shown in blue or bright green, respectively.

To profile the metabolite differences of amino acid, carbohydrate, and lipid metabolism between HL and LL groups, partial least squares-discriminate analysis (PLS-DA) was used (Fig. 2). Leave-one-out cross-validation was applied to validate the model. In colostrum, the Q^2^ of the models were 0.01, 0.44, and 0.10 for amino acid, carbohydrate, and lipid metabolisms, respectively. In contrast, the values were 0.40, 0.08, and 0.05 in mature milk, respectively. The Q^2^ values below 0.3 were considered to be invalid models. Thus, only two models, i.e., amino acid metabolism in mature milk (Fig. 2B, R^2^ = 0.45, Q^2^ = 0.40) and carbohydrate metabolism in colostrum (Fig. 2C, R^2^ = 0.70, Q^2^ = 0.44), were further used to find differential metabolites.

Student’s t-test was used to find differential metabolites in colostrum and mature milk between the two performance groups. The *P*-values were adjusted by applying the Bonferroni correction. To further confirm the biomarkers that may reflect the sows’ metabolic status and the piglets’ nutritional requirements, *P*-value and variable importance in projection (VIP) values calculated by PLS-DA assay were considered at the same time (Table 3). In colostrum, glucuronic acid lactone, mannitol, inositol, inositol, histamine, and allantoin showed significant differences between the two groups (P < 0.05). In mature milk, the level of creatine, glutamate, glutamine, 4-hydroxyproline, alanine, asparagine, and glycine were significantly higher in the HL group (*P* < 0.05), while the levels of arginine and cholesterol were lower in the HL group (*P* < 0.05) (Table 3).

**Table 3:** Differential metabolites between HL and LL groups in colostrum or mature milk.

		HL	LL	*P*-Value	Fold Change	VIP
Colostrum	Glucuronic acid lactone	0.0002	0.0001	0.020	2.00	2.27
	Mannitol	0.0005	0.0018	0.042	0.28	1.42
	Inositol	0.4539	0.6084	0.034	0.75	0.85
	Histamine	2.3986	0.6765	0.042	3.55	0.50
	Allantoin	0.2156	0.0929	0.040	2.32	0.14
Mature milk	Galacturonic acid	0.002	0.008	0.017	0.24	1.83
	Creatine	474.676	346.370	0.009	1.37	3.40
	Glutamine	243.899	178.482	0.007	1.37	2.29
	4-Hydroxyproline	56.446	31.040	0.001	1.82	1.70
	Glutamate	161.169	125.929	0.037	1.28	1.57
	Arginine	22.898	30.828	0.034	0.74	1.48
	Alanine	38.439	27.439	0.009	1.40	1.03
	Asparagine	18.862	13.666	0.005	1.38	0.67
	Glycine	5.341	3.812	0.015	1.40	0.34
	Allantoin	0.148	0.081	0.001	1.82	0.10
	Cholesterol	0.005	0.007	0.013	0.83	2.11

HL: The peak area of target metabolites/internal standard in high lactation performance group; LL: The peak area of target metabolites in low lactation performance; *P*-value: The *P*-value of Student’s t-test; FC: fold change, peak area in HL group/ peak area in LL group. If FC > 1, it means the concentration of metabolites in HL group are higher than LL group, and vice versa. VIP: variable importance in projection (VIP) value calculated by PLS-DA assay.

## Discussion

In this study, high and low lactation performance of sows was characterized according to the weight gain of piglets during the lactation period (D1–D21) following previously published definitions^1^. The milk composition difference between HL and LL groups may reflect the physiological differences of sows and nutritional requirements of piglets.

The concentration of protein, fat, and lactose in sow milk were analysed. We observed that fat tended to be higher in the HL group of colostrum samples, while lactose was significantly higher in the HL group of mature milk samples. Milk fat and lactose are the main digestible energy sources for piglet growth. For neonatal piglets, fat in colostrum is the main energy source for preventing neonatal death, supplying approximately 50% of the required energy. In contrast, lactose is the main energy source in mature milk for sucking piglets^28,29^. Our study confirmed that the concentration of main energy sources is higher in HL group, either in colostrum or in mature milk. The concentration of fat in sow milk increases within 24 h postpartum, which suggests the high requirement of fat for the piglets at the first day of birth. Conversely, the concentration of lactose increases through the whole lactation period, which suggests a continuous rising requirement of lactose by piglets^30^. High fat levels in colostrum and high lactose levels in mature milk may beneficial to piglet growth, suggesting a potential application for the development of functional dietary additives for piglets.

For sows, high levels of catabolism during lactation may lead to weight loss, decrease in back-fat thickness, metabolic disorders, or other diseases^31^. According to the previous studies, the female animals in lactation suffered from insulin resistance due to the drastic changes in lipolysis and energy metabolism^32,33^. Moreover, the female animals may suffer from such diseases as mastitis^34^. It is important to focus on the health and metabolic status of sows and to identify biomarkers that may relate to lactation performance. Our data showed that in the colostrum, mannitol was significantly higher in the LL group than in HL group. Mannitol is one of the most common sugar alcohols in plant species, algae, fungi and lichens but not for mammals^35,36^. Milk mannitol may come from feed or intestinal microbiota and can be considered to be the marker for the permeability of intestine^37^. These results suggested that the sows in the LL group might suffer from intestinal barrier injury, potential mastitis, or bacterial infection. Our data also showed that the glucuronic acid lactone was significantly higher in the HL group. Glucuronic acid lactone is an important metabolite involved in uridine diphosphate (UDP) glucose metabolism and essential for mammals to metabolize drugs, immunoregulation and hematopoiesis^38–40^. The data suggests that the higher concentration of glucuronic acid lactone in HL group may benefit for liver health and anti-infection of piglets.

**Figure 3:**
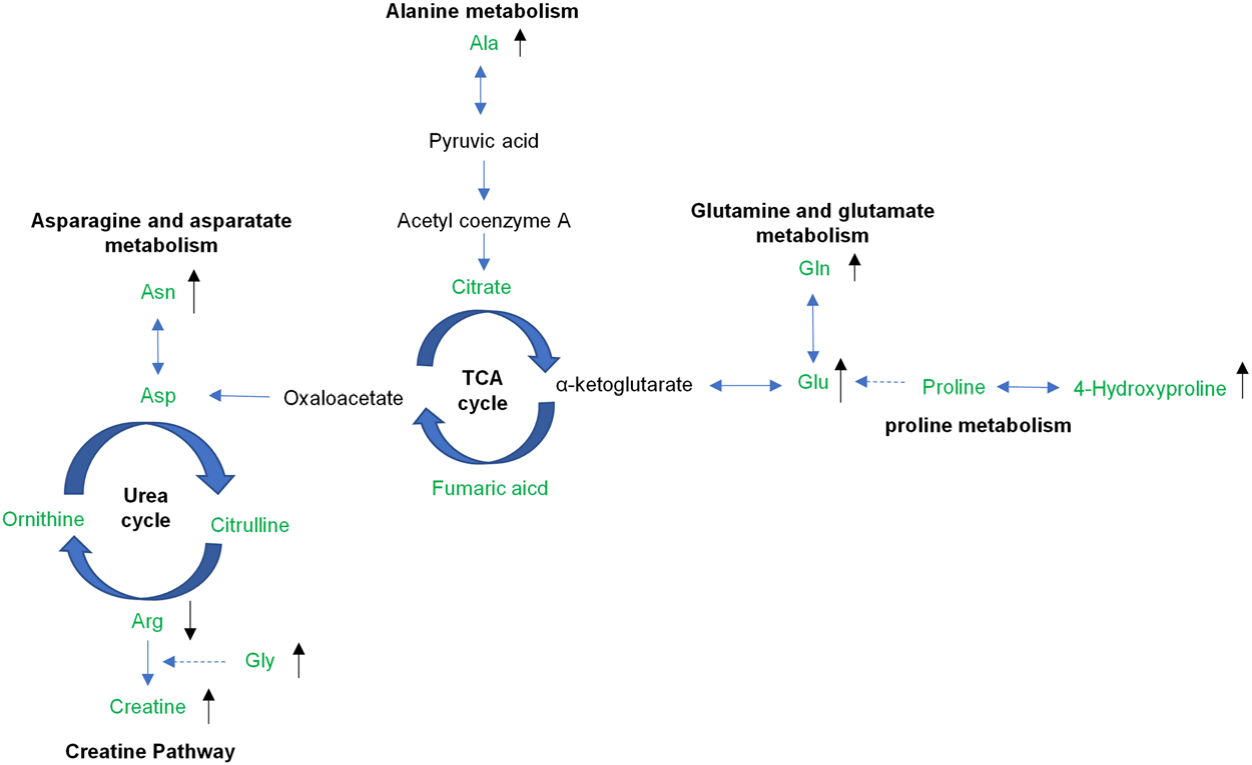
Amino acid and derivates change in mature milk of sows. The metabolites marked in green were detected by GC-MS/LC-MS platform. The differential metabolites are shown with arrows.

Mature milk composition can reflect the nutritional requirements of piglets. Our data showed that the levels of creatine, glutamate, glutamine, and four additional metabolites belonging to amino acids were significantly higher in the HL group for mature milk (Fig. 3), suggesting that the higher lactation performance may be associated with increased levels of amino acids and/or their derivates.

Glutamate and glutamine are important energy source for intestinal cells, and most of the glutamine and glutamate are consumed in first-pass metabolism in the intestines^39^. Glutamate can transform into α- ketoglutarate acid, which is later oxidized to produce ATP to maintain the proliferation of cells^40,41^. Glutamate and glutamine are also important for the development of piglet intestines^42^. Piglets often suffer from diarrhoea and intestinal barrier disruption. The diarrhoea may induce gut permeability, villus atrophy, epithelial lymphocyte infiltration, and other damages to intestinal structure. According to the previous studies, glutamate and glutamine are important for intestinal mucosa morphology development. Glutamine-deprived animals display sloughing of microvilli and decreased actin cores^43^. Furthermore, it has been demonstrated that glutamate and glutamine improve intestinal permeability and increase the expression of intestinal tight junction protein *in vivo* and *in vitro*^44,45^. Additionally, glutamine inhibits oxidative stress-induced apoptosis of intestinal cells and maintains the homeostasis of the gastrointestinal tract^46^. In this study, we found that the levels of glutamine and glutamate in the HL group are significantly higher than those in the LL group, suggesting that the piglets in HL group may have more advantages for intestinal barrier construction, intestinal cell proliferation, and immune regulation.

Glutamate and glutamine are also involved in arginine and proline metabolism. Arginine is synthesized from citrulline with the help of argininosuccinate (ASS) and argininosuccinate lyase (ASL), while citrulline is derived from ornithine via catabolism of proline or glutamate^47^. For young mammals, proline is an essential amino acid that is remarkably deficient in milk^48,49^. The endogenous synthesis of glycine is insufficient in piglets, and supplementation is required^50^. Glutamine is also an essential compound for creatine synthesis along with arginine. Creatine is a major energy source for phosphorylation in muscle tissue^51^ and fat metabolism for mammals^52^. Furthermore, in recent years, creatine has been found to be a protective substance for the nervous system, which can be absorbed and stored in brain cells: oral creatine may benefit brain injury and other neurological diseases^53^.

Arginine is an essential part of the synthesis of biological molecules, such as nitric oxide (NO), polyamines, and creatine. Arginine promotes muscle protein synthesis through the mTOR signalling pathway^54^ and regulates immune response^55^. For sows, the ability to produce milk depends on the health and growth of the mammary glands. According to the previous studies, arginine, a common substrate in generate nitration oxide and polyamines, may regulate the angiogenesis, protein synthesis, and mammary gland development, thereby improving the lactation performance in mammals^56^. In this study, we found that arginine was significantly lower in the HL group, which suggests that under normal circumstances, the mammary gland may primarily use arginine in development and may need to be supplemented in the diet.

Asparagine is also an important amino acid for intestinal energy supply, development, and immune functions. It is reported that asparagine can relieve lipopolysaccharide (LPS)-induced injury and improve intestinal morphology through regulation of the AMPK signalling pathway and improvement of the energy status of the piglets. Asparagine can also be transformed into aspartic acid, which is an essential element for arginine synthesis along with citrulline (Fig. 3). Our data showed that in mature milk, the level of asparagine is significantly higher in the HL group than in LL group, which may be attributable to the improved intestinal functions regulated by asparagine.

## Conclusions

Using GC-MS and LC-MS techniques, significant differences between the metabolites and metabolic pathways in the colostrum and mature milk of sows with high or low lactation performance were observed. Among these metabolic pathways, amino acid metabolism and carbohydrate metabolism are highly correlated with lactation performance of sows, while lipid metabolism shows weak correlation. Up-regulated glutamine, glutamate, alanine, and asparagine are the key metabolites correlated with high lactation performance. This property may be attributed to the benefits of these amino acids for intestinal health, muscle development, and energy status of piglets. The higher concentration of lactose is also a key factor related to high lactation performance, which suggests that energy supply is also important for lactation performance. Besides, increased level of mannitol and lowered level of glucuronic acid lactone in the colostrum are related to low lactation performance. This study may help researchers to understand the key factors in improving the lactation performance and provide useful information for animal nutrition.

## Methods

### Animals and sample collection

The experiment was performed according to the Chinese guidelines for animal welfare and approved by the Institutional Animal Care and Use Committee of the South China Agricultural University (Guangzhou, China). Thirty Yorkshire sows with an average parity of (4.0 ± 1.2) were divided into high and low lactation performance groups (each containing 15 sows), as determined by the piglet weight gain during the lactation period (D1–D21) (Table 4). The weaned piglets with higher piglet weight gain (4.38 ± 0.64 kg) were defined as the high lactation performance group (HL) while the group with lower piglet weight gain (3.24 ± 0.79 kg) was defined as the low lactation performance group (LL). Piglets were weaned at an age of 21 days. After farrowing, the litter size was adjusted within 24 h after birth and weighed by cross-fostering piglets, ensuring that each sow nursed a similar number of piglets, and the average body weight of piglets had no significant difference. The colostrum was collected within 4 h after the initiation of farrow. The mature milk samples were collected at 18th day after parturition. The milk samples were split into 1.5 mL centrifuge tubes and later stored at −80 °C for further testing.

**Table 4:** Lactation performance of sows in HL and LL groups.

Groups	Sow number	Average body weight (kg) after cross-foster	Average body weight (kg) at weaning	Piglet weight gain (kg)
HL	15	1.81 ± 0.19	6.19 ± 0.66	4.38 ± 0.64
LL	15	1.70 ± 0.21	4.95 ± 0.79	3.24 ± 0.79
*P*-Value		0.10	<0.001	<0.001

HL: High lactation performance group; LL: low lactation performance group; Piglet weight gain = Average body weight at weaning - Average body weight after cross-foster.

### Chemical and reagents

Methanol (HPLC-grade), pyridine (HPLC-grade), methoxyamine hydrochloride, margaric acid N,O-bis (trimethylsilyl) trifluoroacetamide (BSTFA) containing 1% trimethylchlorosilane (TMCS) (V:V) were obtained from Sigma-Aldrich. Acetonitrile (HPLC-grade) and formic acid were obtained from Merck. 2-morpholinoethanesulfonic acid (MES) was obtained from J&K Scientific Ltd. Methoxyamine hydrochloride was dissolved in pyridine. Margaric acid and MES were dissolved in methanol and used as the internal standard for GC-MS and LC-MS, respectively. The concentrations of margaric acid and MES were 60 mM and 400 μM, respectively.

### Chemical analysis

The milk composition (fat, lactose, protein and total solid) was determined using a near infrared reflectance spectroscopy instrument (Milk-Scan 134 A/B). Prior to analysis, colostrum and milk were separated by centrifugation at 3000 × g at 4 °C for 20 min.

### Preparation of samples for GC-MS analysis

300 μL milk samples were centrifuged at 4 °C, 3000 g for 10 min to remove the fat. Next, 300 μL methanol and 5 μL margaric acid were added to 100 μL of the skimmed milk. After vortexing, the mixtures were held at 4 °C for 20 min to precipitate protein, and then were dried by nitrogen flow. Next, 100 μL o-methyl hydroxylamine hydrochloride pyridine solution was added and the solution was incubated at 37 °C for 90 min. Subsequently, 100 μL N,O-bis (trimethylsilyl) trifluoroacetamide (BSTFA) derivatization agent containing 1% trimethylchlorosilane (TMCS) (V:V) was added to each sample, followed by incubated at 70 °C for 60 min. The samples were later analysed by GC-MS.

### Preparation of samples for LC-MS analysis

300 μL milk samples were centrifuged at 4 °C, 3000 g for 10 min to remove the fat. Next, 2-morpholinoethanesulfonic acid (MES, internal) and 300 μL acetonitrile was added to 100 μL of the skimmed milk, placed at −20 °C for 20 min, and then centrifuged at 3000 g for 10 min to precipitate protein. The samples were subsequently analysed by LC-MS.

### Identification and quantification of compounds in samples

All GC-MS analyses were performed using a gas chromatography instrument (Shimadzu GC2010, Kyoto, Japan) coupled to a mass spectrometer (GC-MS-QP2010, Kyoto, Japan). The system was equipped with a DB-5 capillary column (Agilent Cat No 122-5033, 30 m × 0.25 mm I.D. df = 1.00 μm).

The injection of a 1 μL aliquot was run in splitless mode at an injector temperature of 280 °C. The oven temperature was 85 °C, which was held for 5 min followed by a ramp of 5 °C per min to 285 °C, held for 5 min, and followed by an increase of 20 °C to 305 °C, which was held for 7 min.

The ion source temperature was 230 °C. Ions were generated using a 70 eV electron beam. The data were collected after an acquisition delay of 5.2 min, 30 spectra s^−1^ were recorded over the mass range 33–1000 m/z with unit resolution. The total flow was 17 mL/min, and the column flow was 1.1 mL/min. The ion source temperature was 200 °C.

A sample aliquot of 5 μL aliquot was injected on the LC-MS instrument (Shimadzu LC-MS 8050, triple quadrupole mass spectrometer) using the ESI ion source with a Discovery HS F5–3 (2.1 mmI.D. × 150 mmL, 3 µm) column. Solvent A was 0.1% formic acid in water (V: V), solvent B was 0.1% formic acid in ACN (V: V) and the ladder elution mode was used. The gradient programme is as shown in Table 5, and the flowrate was 0.25 mL/min. The column temperature was 40 °C.

**Table 5:** Gradient programme for LC-MS analysis.

Step	Time (min)	Solvent A (%)	Solvent B (%)
0	0.0	100	0
1	2.0	100	0
2	5.0	75	25
3	11.0	65	35
4	15.0	5	95
5	20.0	5	95
6	20.1	100	0
7	25.0	100	0

The multiple reaction monitoring (MRM) model was used to identify the metabolites. The operating parameters for electrospray ionization (ESI) source were as follows: 3.0 L/min nebulizer gas flow rate, 10.0 L/min heater gas flow, 400 °C interface temperature, 250 °C desolvation temperature, 400 °C heat block temperature.

To ensure the reliable of the data, a quality control (QC) sample was prepared by mixing 10 μL of each testing sample. The QC sample was analysed five times before sample testing. Additionally, after ten samples were tested, the QC samples were tested twice again to ensure the instrument was stable over the data acquisition process.

### Data analysis

The peak area of the detected components was normalized by the peak area of internal standard. Partial least squares discriminant analysis (PLS-DA) was performed on the treated data using SIMCA-P 13.0 software. The data scale conversion mode used in mature milk and colostrum samples was processed using Pareto Scaling. The outliers were detected and removed using a Monte-Carlo method^57^. Monte-Carlo simulations were used to randomly generate sub-datasets to build PLS-DA sub-models. The rest of the samples were used as test sets. The prediction errors of each sample were analysed statistically and the outliers were detected according to a 3-sigma rule to minimize the risk of removing normal samples. Variable importance in projection (VIP) was also considered to screen out the discriminant metabolites.

The data were analysed using the student’s t-test with the Bonferroni correction. The metabolites with P < 0.05 were considered to have significant difference between the two groups. The discriminant metabolites (*P* < 0.05 and VIP > 1) were considered as the potential biomarkers for high or low lactation performance. The metabolites with significant difference were identified using the Kyoto Encyclopedia of Genes and Genomes (KEGG) database and the relative metabolism pathways were obtained.

### Ethics statement

The experiment was performed according to the Chinese guidelines for animal welfare and approved by the Institutional Animal Care and Use Committee of the South China Agricultural University (Guangzhou, China).

## Electronic supplementary material

Supplementary Information
