# Supplementary material for: Metabolomic Profiles Reveal Potential Factors that Correlate with Lactation Performance in Sow Milk
Source: Sci Rep. 2018 Jul 16;8:10712. doi: 10.1038/s41598-018-28793-0 (PMC6048051; doi:10.1038/s41598-018-28793-0)
Supplement: Supplementary file 1 — Supplementary Information [file 41598_2018_28793_MOESM1_ESM.pdf]

Metabolomic Profiles Reveal Potential Factors that Correlate with Lactation Performance in Sow Milk

Chengquan Tan[1], Zhenya Zhai[1], Xiaojun Ni[1], Hao Wang[1], Yongcheng Ji[1], Tianyue Tang[1], Wenkai Ren[1,2], Hongrong Long[1], Baichuan Deng[1], Jinping Deng[1] & Yulong Yin[1,3]

[1]Guangdong Provincial Key Laboratory of Animal Nutrition Control, Institute of Subtropical Animal Nutrition and Feed, College of Animal Science, South China Agricultural University, Guangzhou 510642, P.R. China

[2]Jiangsu Co-Innovation Center for Important Animal Infectious Diseases and Zoonoses, Joint International Research Laboratory of Agriculture and Agri-Product Safety of Ministry of Education of China, College of Veterinary Medicine, Yangzhou University, Yangzhou, China

[3]National Engineering Laboratory for Pollution Control and Waste Utilization in Livestock and Poultry Production, Institute of Subtropical Agriculture, the Chinese Academy of Sciences, Changsha, P.R. China

C. T. and Z. Z. contributed equally to this work. Correspondence and requests for materials should be addressed to B. D. (email: dengbaichuan@scau.edu.cn), J. D. (email: dengjinping@scau.edu.cn) or Y. Y. (email: yinyulong@isa.ac.cn)

GC-MS data

	Retention time (min)	QC1	QC2	QC3
3-Aminopropanoic acid	14.337	0.001482	0.001497	0.001414
3-Hydroxysebacic acid	21.612	0.000382	0.000381	0.000356
4-Hydroxyphenylpyruvic acid	20.017	0.001564	0.001495	0.001532
4-Hydroxyproline	15.625	0.000589	0.000605	0.00066
Allose	19.905, 20.251	0.023094	0.023681	0.024023
Arabinose	17.326	0.000454	0.000187	0.000431
Aspartic acid	15.574	0.000666	0.000727	0.000741
Cholesterol	34.171	0.001702	0	0
Citraconic acid	13.188	0.00015	0.002556	0.002327
Citric acid	19.09, 19.101	0.04059	0.029563	0.029877
Creatinine	16.146	0.010992	0.011242	0.014598
Cystamine	23.229, 23.292	0.000673	0.000762	0.000713
Cysteine	16.019	0.000673	0.000762	0.000713
Elaidic acid	23.015	0.001385	0.001896	0.001875
Erythrulose	14.785,14.907	0.016174	0.019749	0.018285
Fructose	19.219, 19.799, 19.996	0.017153	0.017956	0.018288
Fucose	18.226	0.002156	0.002396	0.002432
Fumaric acid	12.968	0.006678	0.007962	0.006946
Galactosamine	19.935	0.000217	2.42E-05	0.000239
Galactose	19.531,20.363	0.020379	0.010154	0.010163
Galacturonic acid	20.638, 20.75, 20.892, 21.476	0.012212	0.020361	0.020489
Gluconic acid	21.282	0.00207	0.002313	0.001977
Glucose 6-phosphate	24.221,24.434	0.003866	0.003889	0.003951
Glucose	20.108, 20.394, 20.912	0.042821	0.04571	0.046431
Glucuronic acid lactone	19.241	7.19E-05	6.5E-05	9.17E-05
Glucuronic acid	20.577 ,20.801, 20.852, 21.33	0.002853	0.011078	0.011083
Glutamic acid	16.811	0.000186	0.000201	0.000231
Glycerol 3-phosphate	18.429	0.025235	0.009411	0.014512
Glycerol	11.933	0.004108	0.004756	0.004975
Glycine	12.615	0.000534	0.000649	0.000685
Glycolic acid	8.538	0.000158	0.000239	0.000202
Hypotaurine	16.739	0.000337	0.000472	0.000399
Inositol	22.171	0.241582	0.266143	0.27676
Lactic acid	8.292	0.000591	0.000735	0.000822
Linoleic acid	22.971	0.001144	0.001365	0.001312
Lysine	20.23	0.000162	0.000188	0.000231
Lyxose-meto	17.23, 17.302	0.000908	0.000718	0.000862
Malic acid	15.016	0.00014	0.000166	0.000142
Mannitol	20.505	0.000362	0.000465	0.000433
Mannose 6-phosphate	24.332	0.010951	0.010951	0.010951
Mannose	18.474, 20.251	0.011638	0.011536	0.012205
Margaric acid	22.255	1	1	1
Myristic acid	19.305	0.000183	0.000225	0.000239
N-Acetylglutamine	19.616, 20.139	0.0007	0.000679	0.000649
Niacinamide	15.124	0.001241	0.001276	0.001296
Oleic acid	22.989	0.00156	0.002103	0.001962
O-Phosphoethanolamine	16.26, 18.811	0.001849	0.001714	0.00167
Ornithine	19.155	0.006073	0.006388	0.006384
Palmitic acid	21.32	0.004331	0.00521	0.005055
Palmitoleic acid	21.165	0	7.06E-05	7.54E-05
Pantothenic acid	21.009	0.000304	0.000265	0.000274
Phenylalanine	17.027	0.000468	0.000589	0.000496

Phosphoric acid				11.997	0.063243	0.096943	0.100263
Proline				12.483	0.000141	0.000186	0.000205
Propionylglycine				13.246	0.000127	0.000129	0.000119
Psicose	19.198,	19.241,	19.616,	19.745	0.021295	0.011527	0.022135
Pyridoxal				19.884	0.000181	4.35E-05	7.11E-05
Rhamnose		17.887,	18.079,	18.271	0.00469	0.005319	0.005509
Ribose		17.158,	17.266,	17.506	0.001781	0.002283	0.002917
Ribulose 5-phosphate				22.227	0.000483	0.000419	0.00042
Ribulose				17.506	0.0003	0.000369	0.000298
Serine				13.276	0.000767	0.000808	0.000786
Sorbose			19.81,	19.955	0.02008	0.021192	0.021261
Stearic acid				23.158	0.003509	0.004429	0.004166
Tagatose				19.756	0.001288	0.001355	0.001381
Theophylline				20.241	0.001299	0.001315	0.001329
Threitol				15.206	0.003331	0.003488	0.003502
Threonic acid				16.006	0.000341	0.00041	0.00038
Threonine				13.701	0.00178	0.001848	0.000439
Uracil				13.012	0.008937	0.025067	0.02426
Urea				11.404	0.101674	0.122321	0.125777
Valine				11.116	0.00031	0.000317	0.000391
Xylitol				17.91	0.000217	0.000168	0.000131
Xylose				17.242	0.000454	0.000531	0.000431

QC4	QC5	QC6	QC7	QC8	QC9	QC10	QC11	QC12
0.001415	0.001296	0.001482	0.001497	0.001414	0.000929	0.001033	0.000929	0.000999
0.000349	0.000332	0.000382	0.000381	0.000356	0.000297	0.000256	0.000295	0.000288
0.001523	0.001431	0.001564	0.001495	0.001532	0.001186	0.001072	0.001221	0.001186
0.000586	0.000528	0.000589	0.000605	0.00066	0.000318	0.000361	0.000262	0.000334
0.023336	0.02221	0.023094	0.023681	0.024023	0.019977	0.019628	0.020985	0.0209
0.000572	0.000415	0.000138	0.000455	0.000428	0.000193	0.000477	0.000494	0.00047
0.000647	0.00063	0.000666	0.000727	0.000741	0.000743	0.000804	0.000636	0.000721
0.001912	0.001819	0.00194	0.001895	0.001901	0.001787	0.00201	0.0017	0.001629
0	0	0.000118	0.000124	0.000125	0.001787	0.002348	0.001647	0.002376
0.040452	0.040211	0.040969	0.041119	0.040828	0.031136	0.032196	0.034788	0.030983
0.011458	0.010988	0.011524	0.01123	0.011258	0.010454	0.010984	0.015129	0.01426
0.000678	0.000663	0.000673	0.000762	0.000713	0.000488	0.000466	0.000358	0.000435
0.000678	0.000663	0.000673	0.000762	0.000713	0.000488	0.000466	0.000358	0.000435
0.001329	0.001381	0.001327	0.001425	0.001495	0.001924	0.001938	0.001672	0.001806
0.016489	0.016405	0.016232	0.015931	0.016062	0.016918	0.019002	0.017237	0.017132
0.01742	0.017674	0.017325	0.017385	0.017229	0.016274	0.018102	0.018846	0.01813
0.002211	0.002234	0.002199	0.002118	0.002212	0.002158	0.002413	0.002366	0.002478
0.006738	0.006608	0.006764	0.006831	0.00667	0.007008	0.007961	0.006479	0.006565
0.00018	0.000204	0.000213	0.000339	0.000234	0.000183	0.000207	0.000203	0.000204
0.020599	0.020509	0.02052	0.020624	0.020552	0.020789	0.02277	0.022967	0.022746
0.011376	0.011116	0.011232	0.011497	0.011483	0.011249	0.020476	0.020853	0.020184
0.002202	0.002165	0.002159	0.002158	0.00218	0.002134	0.002296	0.002047	0.00212
0.003901	0.003784	0.003874	0.003893	0.003913	0.003311	0.003288	0.00361	0.003445
0.043469	0.043697	0.043093	0.042763	0.043277	0.041284	0.045609	0.04712	0.045709
6.45E-05	9.59E-05	5.31E-05	7.47E-05	7.61E-05	7.38E-05	0.000114	7.56E-05	7.8E-05
0.002933	0.002745	0.002951	0.002966	0.01046	0.010191	0.011255	0.011259	0.010795
0.000178	0.000161	0.000186	0.000201	0.000231	0.000164	0.000145	0.000201	0.00021
0.02779	0.027024	0.026762	0.025887	0.025234	0.016427	0.013039	0.022127	0.017532
0.004105	0.004044	0.004091	0.003988	0.004031	0.004177	0.004548	0.004451	0.004632
0.000538	0.000507	0.000498	0.000474	0.00056	0.000588	0.000632	0.000615	0.000614
0.000107	0.000113	0.000154	0.000148	0.000156	0.000127	0.000218	0.000133	0.000182
0.000348	0.000362	0.000337	0.000472	0.000399	0.000325	0.000298	0.000319	0.000319
0.249071	0.243561	0.250269	0.247626	0.244354	0.236704	0.270491	0.26755	0.267259
0.000559	0.000535	0.000546	0.000541	0.00055	0.000612	0.000682	0.000749	0.000785
0.001084	0.001157	0.001154	0.001095	0.001065	0.001517	0.001413	0.001225	0.001267
0.000177	0.00016	0.000248	0	0.000192	0.000215	0	0.000222	0
0.001143	0.000829	0.000632	0.000904	0.000856	0.000725	0.000955	0.000987	0.000939
0.000144	0.000162	0.000164	0.000149	0.000158	0.00016	0.000149	0.000153	0.000166
0.000393	0.000427	0.00046	0.000399	0.000407	0.00036	0.000446	0.000552	0.00043
0.010951	0.010951	0.010951	0.010951	0.010951	0.010951	0.010951	0.010951	0.010951
0.011761	0.011714	0.011565	0.011426	0.011561	0.010925	0.011678	0.012323	0.012087
1	1	1	1	1	1	1	1	1
0.000226	0.000197	0.000186	0.000178	0.000224	0.000276	0.000241	0.000226	0.00025
0.000833	0.000603	0.000912	0.000631	0.000709	0.000625	0.00067	0.000717	0.000892
0.001352	0.001268	0.001241	0.001276	0.001296	0.001045	0.001031	0.001019	0.001067
0.001496	0.001483	0.001442	0.00156	0.001536	0.002023	0.001978	0.0017	0.001901
0.001891	0.001839	0.001879	0.001861	0.001853	0.001943	0.001974	0.002142	0.003466
0.00619	0.005942	0.006073	0.006388	0.006384	0.004873	0.005057	0.004864	0.005166
0.004285	0.004185	0.004299	0.004294	0.004232	0.004943	0.005381	0.004978	0.004965
0.000103	0	0.0001	8.35E-05	6.27E-05	6.46E-05	0.000128	8.91E-05	9.22E-05
0.000272	0.0003	0.000316	0.000326	0.000305	0.000331	0.000311	0.000327	0.000281
0.0005	0.000474	0.000458	0.000491	0.000455	0.000493	0.000525	0.000503	0.000448

0.065471	0.064652	0.064513	0.063727	0.065006	0.068638	0.082743	0.076819	0.086271
0.000157	0.000131	0.000136	0.000136	0.000172	0.000141	0.000187	0.000182	0.000163
9.05E-05	4.91E-05	8.39E-05	7.27E-05	0.000138	0.000126	0	9.18E-05	0.000114
0.021685	0.02201	0.021262	0.021563	0.021511	0.020039	0.011686	0.022858	0.021973
7.88E-06	5.2E-05	4.45E-05	0.000188	2.97E-05	5.76E-05	7.02E-05	5.05E-05	2.33E-05
0.004875	0.004957	0.00489	0.004694	0.004844	0.004847	0.005419	0.002859	0.005391
0.001837	0.002066	0.000726	0.001975	0.002526	0.002645	0.003025	0.002878	0.002661
0.000566	0.000519	0.000565	0.0005	0.000515	0.000446	0.000516	0.000473	0.000463
0.000319	0.00032	0.000295	0.000281	0.000331	0.000286	0.000347	0.000364	0.000328
0.00075	0.00069	0.000786	0.000793	0.000774	0.00052	0.000507	0.000406	0.00048
0.020399	0.016991	0.020048	0.020347	0.020266	0.015826	0.021263	0.021826	0.021093
0.003339	0.003479	0.003416	0.00346	0.003378	0.003886	0.004273	0.003969	0.003877
0.001387	0.001341	0.00133	0.001337	0.001321	0.001226	0.001382	0.001404	0.001353
0.001312	0.001169	0.001299	0.001315	0.001329	0.00101	0.001022	0.001104	0.001066
0	0	0.003331	0.003488	0.003502	0	0.001683	0	0.001549
0.000332	0.000348	0.000352	0.000358	0.000362	0.000395	0.000413	0.000404	0.000397
0.001806	0.001727	0.00178	0.001848	0.000439	0.001389	0.001409	0.001569	0.001635
0.007807	0.008278	0.008801	0.008713	0.009166	0.015125	0.020784	0.016169	0.02054
0.104226	0.10359	0.105545	0.104372	0.104678	0.10668	0.116101	0.119372	0.119375
0.000328	0.000341	0.000312	0.000292	0.000373	0.000373	0.000381	0.00032	0.000394
0.000165	0.000206	0.000133	0.000263	0.000736	0.000233	0.000237	0.000151	9.86E-05
0.000572	0.000415	0.000494	0.000455	0.000428	0.000532	0.000477	0.000494	0.00047

MHL-1	MHL-2	MHL-3	MHL-4	MHL-5	MHL-6	MHL-7	MHL-8	MHL-9
0.00196	0.000896	0.00159	0.000925	0.002132	0.001378	0.001816	0.001324	0.001394
0.000286	0.000248	0.000176	0.000261	0.000214	0.000243	0.000236	0.00019	0.000201
0.002766	0.001709	0.00071	0.000654	0.001201	0.002556	0.001115	0.000736	0.000703
0.000775	0.000962	0.000669	0.000623	0.000738	0.000336	0.000886	0.000572	0.000397
0.023206	0.017648	0.012845	0.005687	0.011812	0.021784	0.016981	0.008155	0.006311
0.001072	0.001171	0.000638	0.001	0.001172	0.000886	0.000999	0.000922	0.000891
0.001875	0.001443	0.000977	0.001625	0.00171	0.001721	0.000648	0.001226	0.001327
0.005957	0.007536	0.004639	0.005591	0.004927	0.004338	0.004369	0.005206	0.004641
0.000217	0.000251	0.000143	0.000214	0.00028	5.9E-05	8.08E-05	0.000152	0
0.064427	0.068955	0.064715	0.053808	0.078689	0.083795	0.084612	0.068245	0.068967
0.003589	0.017073	0.009734	0.008884	0.007166	0.002025	0.004771	0.008936	0.005983
0.001784	0	0	0	0	0.001993	0.001948	0.002549	0.002697
0.000805	0.000681	0.000326	0.000321	0.000557	0.000709	0.00044	0.000203	0.00021
0.007906	0.007789	0.005442	0.005722	0.006248	0.005704	0.006618	0.005954	0.005447
0.012684	0.01787	0.016452	0.013974	0.015842	0.013188	0.014116	0.013578	0.014503
0.01703	0.015944	0.011384	0.010745	0.011456	0.016375	0.01246	0.012641	0.012485
0.00181	0.001943	0.001014	0.001431	0.000838	0.000968	0.000937	0.001139	0.001699
0.00591	0.005523	0.003076	0.003987	0.009138	0	0.005301	0.006565	0.00453
0.000227	0.000144	9.45E-05	0.000174	0.000127	0.000179	0.000168	0.000154	0.000151
0.136729	0.141212	0.113219	0.114889	0.10987	0.136882	0.106908	0.117733	0.106663
0.013771	0.014347	0.010115	0.016064	0.01145	0.01315	0.012951	0.01434	0.010033
0.003064	0.002247	0.001078	0.002802	0.001395	0.001828	0.001126	0.004372	0.003453
0	0.000497	0	0.000139	0	0.000103	0	0.000271	0
0.135582	0.08657	0.069378	0.023458	0.061297	0.127564	0.060614	0.011732	0.023459
0.00016	0.000248	0.000172	6.74E-05	8.18E-05	0.000144	0.000171	0.000175	0.000104
0.005858	0.005427	0.001383	0.005362	0.00354	0.001851	0.002427	0.006195	0.00657
0.001255	0.001169	0.000973	0.000844	0.000825	0.001192	0.000796	0.00061	0.000654
0.046258	0.06136	0.06974	0.027369	0.046721	0.050202	0.034334	0.031413	0.039294
0.003267	0	0.003119	0	0	0	0	0	0
0.019627	0.021952	0.015413	0.015739	0.012034	0.018314	0.009847	0.010761	0.006261
0.000302	0.000329	0.000237	0.000262	0.000284	0.000221	0.000169	0.000268	0.000229
0.034997	0.01509	0.015311	0.021697	0.035959	0.012888	0.019157	0.027556	0.021107
0.349981	0.419746	0.308271	0.337061	0.283989	0.295885	0.30711	0.305581	0.323007
0.00254	0.003736	0.002203	0.002034	0.00233	0.001666	0.001246	0.001887	0.001248
0.006284	0.006233	0.004755	0.005735	0.005231	0.004758	0.005361	0.006344	0.004416
0	0.001121	0.001255	0.001111	0.000666	0.000688	0.000929	0.000918	0.001095
0.001964	0.002005	0.001149	0.001874	0.001783	0.001244	0.001738	0.001858	0.001883
0.007672	0.006171	0.003111	0.003708	0.012311	0.006633	0.00445	0.00836	0.005787
0.022158	0.01669	0.01232	0.00477	0.011014	0.020719	0.010499	0.000979	0.000947
0.002465	0.002842	0.00026	0	0.001509	0	0	0.001725	0
0.029299	0.023448	0.020904	0.009594	0.017398	0.027968	0.015263	0.013954	0.010282
1	1	1	1	1	1	1	1	1
0.000892	0.00078	0.000533	0.000701	0.000648	0.000598	0.000732	0.000829	0.000637
0.002859	0.00153	0.001661	0.000248	0.001563	0.002221	0.001102	0.00037	0.000249
0.005771	0.003173	0.003308	0.002961	0.003541	0.002855	0.002803	0.002479	0.002209
0.007949	0.007836	0.00559	0.005881	0.006454	0.005883	0.006593	0.005938	0.005525
0.005582	0.006211	0.007594	0.004391	0.004683	0.00582	0.003264	0.003916	0.003685
0	0.000519	0	0	0.000261	0	0	0	0.00029
0.008808	0.008636	0.006599	0.007131	0.007773	0.006391	0.006896	0.007982	0.006727
0.002714	0.001602	0.000681	0.001421	0.001786	0.001073	0.001991	0.002057	0.001979
0.007109	0.009125	0.006158	0.005991	0.007578	0.006851	0.006171	0.006798	0.006308
0.00339	0.005035	0.003853	0.003633	0.003696	0.004054	0.00241	0.00287	0.002516

0.137044	0.161465	0.126401	0.109948	0.120769	0.14373	0.113781	0.122833	0.092287
0.000457	0.001593	0.001566	0.001085	0.001094	0.000517	0.000816	0.000927	0.000767
0.001314	0.0015	0.000724	0.000758	0.001345	0.001487	0.001037	0.000687	0.000856
0.011829	0.011458	0.008467	0.007722	0.008695	0.011635	0.009428	0.009143	0.009219
0	8.38E-05	0	0.000151	0	0	0	0	6.22E-05
0.006789	0.007201	0.004634	0.003715	0.005176	0.006678	0.003808	0.00316	0.003612
0.002587	0.003268	0.004013	0.002845	0.003305	0.002479	0.002263	0.004051	0.002327
0.003452	0.003507	0.002228	0.001595	0.004208	0.002133	0.002789	0.005679	0.002525
0.001141	0.000949	0.000416	0.001047	0.000917	0.000558	0.000528	0.001593	0.000731
0.000488	0.000625	0.000266	0.000324	0.000521	0.000647	0.000487	0.000271	0.000288
0.019211	0.014803	0.010351	0.009891	0.010205	0.01783	0.013516	0.011586	0.013835
0.006511	0.007029	0.00633	0.005307	0.005436	0.005087	0.005296	0.005082	0.004922
0.001247	0.001031	0.000789	0.000719	0.000716	0.001119	0.000866	0.000897	0.00087
0.004274	0.00386	0	0.001913	0.0032	0	0	0.002552	0.002609
0.001194	0.000932	0.000483	0.000577	0.001916	0.001089	0.000717	0.000129	0.000908
0.000407	0.000314	0.00018	0.000909	0.000378	0.000689	0.000649	0.000333	0.000693
0.006616	0.005404	0.00123	0.00438	0.007281	0.006763	0.005058	0.003172	0.004209
0.014788	0.012171	0.01031	0.011134	0.015627	0.006445	0.006957	0.01243	0.008213
0.71606	0.578277	0.461483	0.488226	0.650827	0.705508	0.527483	0.59659	0.51844
0.000535	0.000919	0.000904	0.000572	0.00077	0.000684	0.000535	0.000448	0.000479
0.000225	0.000168	9.14E-05	0.000209	0.000207	0.000175	0.000209	0.000214	0.00021
0.000892	0.000834	0.000511	0.000873	0.00061	0.000359	0.00074	0.000936	0.000991

MHL-10	MHL-11	MHL-12	MHL-13	MLL-1	MLL-2	MLL-3	MLL-4	MLL-5
0.001163	0.000769	0.001501	0.001376	0.001643	0.000996	0.000401	0.000607	0.001634
0.00027	0.000306	0.00023	0.000202	0.000239	0.000288	0.00031	0.000356	0.000252
6.2E-05	0.000842	0.000114	0.000668	0.001205	0.002523	4.11E-06	0.001908	0.000675
0.000387	0.000317	0.000444	0.000454	0.000747	0.000655	0.000181	0.000272	0.000492
0.028624	0.010047	0.008413	0.020539	0.018084	0.021034	0.007697	0.017532	0.024662
0.000971	0.001377	0.001085	0.000735	0.000738	0.001504	0.001106	0.001221	0.000929
0.001159	0.000744	0.00053	0.0004	0.002188	0.001251	0.001355	0.000582	0.002096
0.004839	0.006138	0.006386	0.004948	0.006121	0.004825	0.007468	0.006283	0.005671
0.000124	0.000106	0	0	0.000163	0.000157	0.000181	0	0
0.075407	0.082263	0.049819	0.057741	0.076193	0.076663	0.055684	0.098362	0.07178
0.005532	0.004359	0.002598	0.006487	0.014536	0.008643	0.005544	0.007061	0.003431
0.003555	0.002034	0	0.000756	0	0	0.003382	0	0.001753
0.000479	0.00055	0.000249	0.000201	0.000339	0.000602	0.000268	0.000787	0.000364
0.006111	0.005446	0.00622	0.006675	0.005863	0.005907	0.006749	0.006102	0.00677
0.01389	0.014116	0.01246	0.011692	0.016204	0.017188	0.015002	0.014845	0.013948
0.009853	0.014273	0.011136	0.01111	0.0169	0.016987	0.021473	0.014034	0.014312
0.000972	0.002838	0.001326	0.000946	0.00108	0.001036	0.002441	0.001597	0.001122
0	0.004842	0.01229	0.004607	0.006847	0.006988	0.005864	0.00564	0.006517
0.000115	0.000208	0.000155	0.000155	0.000233	0.000205	0.000236	0.000168	0.000185
0.109767	0.112486	0.09653	0.006594	0.119361	0.155597	0.091254	0.115092	0.106973
0.013591	0.015569	0.015891	0.014709	0.017669	0.018685	0.020433	0.01808	0.020953
0.003545	0.004567	0.002606	0.001328	0.003923	0.002564	0.00457	0.001579	0.001041
0	0	0	0	0.000265	0.000479	0	0.00135	5.84E-05
0.036425	0.038702	0.028118	0.027587	0.064988	0.12642	0.020392	0.092417	0.03625
6.78E-05	6.1E-05	0.000166	9.86E-05	0.000145	0.000225	0.000168	0.000227	0.000245
0.002246	0.004911	0.005749	0.004855	0.009041	0.005816	0.006906	0.004334	0.007644
0.000511	0.000598	0.000719	0.000611	0.000955	0.000605	0	0.000451	0.00104
0.046387	0.036677	0.039311	0.037192	0.0343	0.048742	0.033441	0.050673	0.042781
0	0	0	0	0	0	0	0	0.001892
0.009279	0.014566	0.020454	0.012768	0.019946	0.011606	0.005626	0.010376	0.013338
0.000227	0.000292	0.000368	0.000347	0.000286	0.000345	0.000247	0.000218	0.000235
0.021413	0.016211	0.018184	0.0247	0.025266	0.018631	0.008671	0.010312	0.021816
0.327556	0.425844	0.383127	0.301742	0.477487	0.390733	0.367456	0.431631	0.408698
0.001975	0.001813	0.002319	0.002544	0.001547	0.002453	0.003109	0.002286	0.001926
0.00551	0.004741	0.004687	0.005284	0.00585	0.006063	0.005299	0.005789	0.006046
0	0.001311	0.000409	0.000525	0.000932	0.000968	0.00078	0.000753	0.000501
0.001641	0.002457	0.002067	0.001198	0.001499	0.002228	0.002116	0.002093	0.00168
0.011686	0.004678	0.005124	0.00419	0.008978	0.009654	0.006015	0.008476	0.007397
0.007172	0.0023	0.002813	0.005672	0.011152	0.019911	0.006295	0.016753	0.003336
0	0	0	0	0	0.002724	0	0.005462	0
0.033842	0.014174	0.014634	0.013318	0.016892	0.028645	0.010753	0.02518	0.030632
1	1	1	1	1	1	1	1	1
0.000622	0.000562	0.000709	0.00086	0.000633	0.00078	0.000735	0.000678	0.000912
0.000678	0.00065	0.000754	0.000936	0.001102	0.002628	0.000274	0.00158	0.00057
0.002667	0.002766	0.004757	0.004014	0.002609	0.004788	0.003176	0.003531	0.003235
0.006302	0.005588	0.006315	0.006424	0.005923	0.005932	0.006879	0.006404	0.00715
0.004784	0.003806	0.003558	0.004299	0.006607	0.005448	0.002836	0.007037	0.005019
0	0	0.000324	0.000369	0.000359	0	0	0	0
0.007307	0.006964	0.007081	0.007006	0.007877	0.007678	0.008179	0.006765	0.007129
0.00188	0.001285	0.001731	0.001483	0.001298	0.001768	0.001288	0.001367	0.002779
0.006472	0.007281	0.006761	0.007672	0.00787	0.00678	0.004662	0.007824	0.007194
0.001956	0.004142	0.001826	0.003515	0.003998	0.004059	0.004385	0.003263	0.002344

0.142987	0.108999	0.129772	0.122507	0.158675	0.159784	0.079506	0.182032	0.112063
0.00051	0.00125	0.000424	0.000329	0.001175	0.001241	0.000925	0.001363	0.000603
0.0006	0.000976	0.001001	0.000827	0.001845	0.001066	0.000589	0.001377	0.000874
0.007788	0.010706	0.007859	0.008122	0.011839	0.011887	0.01418	0.010792	0.010169
0	0.000125	0	0	0	0	7.26E-05	4.89E-05	0
0.004127	0.006136	0.003703	0.004087	0.003898	0.005669	0.004924	0.006031	0.004606
0.002637	0.003237	0.001593	0.001723	0.002279	0.004124	0.002567	0.0026	0.002539
0.005478	0.00142	0.004231	0.003219	0.004737	0.003904	0.00163	0.002957	0.004089
0.000785	0.000788	0.001701	0.000821	0.00118	0.000831	0.001317	0.000521	0.00072
0.000245	0.000409	0.000388	0.00027	0.000751	0.000431	0.000237	0.000536	0.000358
0.008579	0.015847	0.010343	0.010155	0.015684	0.015794	0.020602	0.01248	0.015714
0.00549	0.004861	0.00506	0.005412	0.006954	0.005705	0.006191	0.005541	0.004882
0.000644	0.000898	0.00078	0.000765	0.001257	0.001193	0.001575	0.000959	0.000968
0.002766	0.001875	0	0.001084	0.003256	0.003513	0.002998	0.002089	0
6.53E-05	8.76E-05	9.19E-05	0.000687	0.001493	6.55E-05	0.001032	0.001348	0.001175
0.000504	0.000905	0.000686	0.000845	0.000285	0.000362	0.001026	0.000691	0.000787
0.003158	0.004363	0.004749	0.003909	0.008785	0.004759	0.001226	0.008906	0.00457
0.010646	0.013201	0.005588	0.009859	0.005851	0.011889	0.017189	0.007154	0.007921
0.598534	0.635856	0.375723	0.327914	0.672621	0.69577	0.535808	0.984119	0.760293
0.000479	0.001024	0.000461	0.000236	0.001114	0.000721	0.000645	0.001745	0.00074
0.00014	0.000172	0.000113	0.00013	0.000261	0.000264	0.00017	0.000151	0.000153
0.00067	0.001081	0.000981	0.000463	0.000761	0.000724	0.00101	0.000872	0.000751

MLL-6	MLL-7	MLL-8	MLL-9	MLL-10	MLL-11	MLL-12	CLL-1	CLL-2
0.000809	0.001301	0.00043	0.001058	0.001081	0.000426	0.001135	0	0
4.05E-05	0.000333	0.000268	0.000332	0.000359	6.13E-05	0.000345	0.000314	0.00035
0.00052	7.52E-05	0.000571	6.59E-05	0.000654	0.001166	5.87E-05	0.000999	0.002824
0.000285	0.000351	0.000124	0.000216	0.000285	0.000147	0.000225	0	0
0.006977	0.010776	0.008927	0.004185	0.023463	0.005714	0.007116	0.028937	0.033118
0.000886	0.001427	0.001354	0.000728	0.00091	0.001642	0.00084	0.000658	0.001098
0.000588	0.000518	0.000247	0.001491	0.00032	0.000648	0.001965	0	6.27E-05
0.007	0.008425	0.006418	0.006167	0.006521	0.007595	0.005677	0.007986	0
0.000111	0	0.000188	0	0	6.31E-05	5.37E-05	0.000112	0.000408
0.074908	0.036048	0.040303	0.068482	0.060105	0.048736	0.058642	0.115333	0.092568
0.004241	0.014242	0.015536	0.015674	0.002122	0.01237	0.02355	0.017189	0.020269
0.002387	0.001448	0.001366	0.001873	0	0.002066	0.001785	0.001691	0.001629
0.000298	0.000413	0.000436	0.000475	0.0002	0.000297	0.000506	0.000242	9.63E-05
0.006041	0.00797	0.005603	0.007052	0.00516	0.006616	0.006168	0.002276	0.001996
0.014125	0.014593	0.013894	0.013476	0.004902	0.015056	0.015279	0.016277	0.01602
0.013196	0.017921	0.009229	0.009077	0.012244	0.018752	0.010778	0.014925	0.044001
0.001716	0.002576	0.002954	0.001104	0.001084	0.002557	0.001804	0.005503	0.003551
0.005637	0.005753	0.000861	0.006702	0.004431	0.007335	0.006967	0.025468	0.010462
0.000151	0.000186	9E-05	0.000115	0.000115	0.000224	0.000116	0.000482	6.44E-05
0.10755	0.09881	0.009956	0.005731	0.097918	0.079783	0.008061	0.024872	0.02394
0.016428	0.027939	0.036782	0.026039	0.019229	0.02823	0.018559	0.029421	0.020605
0.002143	0.002128	0.001953	0.002641	0.002302	0.004642	0.001593	0.011857	0.006301
0.00026	0.001055	0	0.000617	0	0.001073	0	9.43E-05	3.53E-05
0.023153	0.013097	0.037972	0.009282	0.031852	0.016342	0.02224	0.110501	0.10547
0.000119	0.000193	0.000444	0.000124	0.000193	0.000152	6.73E-05	0.000592	0.000106
0.003637	0.01299	0.026214	0.014174	0.001911	0.016102	0.00853	0.015154	0.004679
0.000537	0.000481	0.000164	0.000529	0.00056	0.000338	0.000832	0	0
0.037722	0.045116	0.078889	0.072365	0.021184	0.030033	0.038202	0.107114	0.088698
0	0.00491	0.003105	0	0	0	0.004102	0.007284	0.006045
0.010247	0.009673	0.002852	0.006556	0.01823	0.00647	0.008842	0.000212	0.000273
0.000363	0.000338	0.00032	0.000459	0.000245	0.000363	0.000327	0.000217	0.000166
0.019579	0.021519	0.017149	0.016336	0.022004	0.01369	0.006069	0	0
0.358689	0.358946	0.317814	0.367641	0.359013	0.342024	0.405916	0.747924	0.612239
0.003017	0.004808	0.001509	0.002882	0.003619	0.004992	0.004979	0.000406	0.000548
0.005758	0.004681	0.00459	0.005734	0.004255	0.00519	5.26E-05	0.002129	0.001882
0.000994	0.001002	0.001439	0	0	0.000809	0.001722	0.000278	0.000146
0.001677	0.002277	0.002265	0.001455	0.00172	0.002698	0.001565	0.001316	0.002197
0.006675	0.002728	0.002139	0.020552	0.00518	0.007741	0.004655	0.00036	0.000588
0.001246	0.001096	0.000459	0	0.007682	0.001236	0.003084	0.008685	0.000863
0.003237	0.000891	0	0.003168	0	0.004561	0	0.000188	0.000131
0.013869	0.010357	0.01524	0.012681	0.027876	0.008885	0.010709	0.035076	0.029782
1	1	1	1	1	1	1	1	1
0.0007	0.000769	0.000601	0.00112	0.000475	0.00081	0.00058	0.000176	0.000207
0.000541	0.000329	0.000679	0.00063	0.00107	0.000592	0.000609	0.001653	0.001555
0.002483	0.004336	0.002708	0.002806	0.003629	0.003178	0.003267	0.000978	0.000523
0.006015	0.008337	0.005429	0.006692	0.005622	0.006765	0.006592	0.002423	0.002061
0.004142	0.005284	0.003091	0.00585	0.00244	0.004233	0.005621	0.011227	0.010022
0.000152	0.000477	0	0.00039	0	0	0.000532	0	0
0.007933	0.009087	0.006558	0.009191	0.00636	0.008667	0.007392	0.00591	0.006011
0.001528	0.001201	0.001372	0.003203	0.001052	0.00233	0.001495	0	0
0.006192	0.00389	0.006223	0.006325	0.005561	0.003864	0.004888	0.000751	0.001153
0.003632	0.004676	0.002163	0.003827	0.000181	0.003418	0.004033	0.000512	0.000343

0.118497	0.106626	0.121846	0.194556	0.102093	0.100452	0.087625	0.271485	0.243399
0.000873	0.001018	0.000746	0.000871	0.000132	0.000619	0.001386	0.000227	0.000193
0.001234	0.001248	0.000396	0.000984	0.000875	0.000699	0.000956	0.000111	8.34E-05
0.009685	0.011691	0.011501	0.00763	0.008759	0.012364	0.008008	0.011516	0.053767
0	0	0.000116	0	0	4.45E-05	0	1E-04	0.000101
0.005055	0.005443	0.003704	0.002342	0.004028	0.005017	0.003741	0.007265	0.005058
0.002337	0.002829	0.003123	0.002292	0.002545	0.004809	0.001933	0.001858	0.003473
0.004173	0.000862	0	0.00273	0.003577	0.001461	0.001132	0.00115	0.000632
0.000793	0.000691	0.000203	0.001196	0.001028	0.001354	0.000811	0.000258	0.000147
0.0005	0.000481	0.000125	0.000358	0.000366	0.000264	0.000367	0	0
0.011999	0.019976	0.008426	0.008693	0.013213	0.017789	0.00965	0.015346	0.050196
0.006104	0.006838	0.004494	0.00595	0.005241	0.006617	0.005645	0.006645	0.006575
0.000914	0.001308	0.000647	0.000531	0.000855	0.001335	0.000706	0.000979	0.00342
0.003021	0.001721	0	0.002292	0	0.002842	0.001725	0	0
4.99E-05	8.15E-05	0.000299	0.003216	0.000843	0.000127	0.000738	0	5.44E-05
0.001034	0.001086	0.002078	0.000397	0.000683	0.000734	0.001276	0.001849	0.000983
0.002933	0.003187	0.000626	0.003639	0.006153	0.001448	0.002099	0	0
0.019034	0.010428	0.010743	0.010829	0.007888	0.017542	0.010002	0.002348	0.004961
0.414516	0.415185	0.298619	0.606338	0.360785	0.508236	0.365094	0.475985	0.293121
0.000713	0.00105	0.000322	0.00092	0.000255	0.000579	0.00129	0.000374	0.000162
0.000138	0.000169	9.57E-05	0.000117	0.000149	0.000168	2.86E-05	8.01E-05	8.34E-05
0.000791	0.00085	0.000912	0.000727	0.00081	0.001056	0.000724	0.000658	0.001098

CLL-3	CLL-4	CLL-5	CLL-6	CLL-7	CLL-8	CLL-9	CLL-10	CLL-11
0	0.000133	0	0.000155	0	0	0	0	0
0.000317	0.000575	0.00041	5.74E-05	9.54E-05	0.000654	9.82E-05	0.001155	0.000513
0.001351	0.001455	0.00138	0.000974	0.007401	0.000214	0.004859	4.81E-05	0.001901
0	0	0	0	0	0	0	0	0
0.012039	0.033445	0.024645	0.013785	0.032677	0.025166	0.030235	0.038352	0.026259
0.000521	0.000527	0.001432	0.001614	0.001178	0.001751	0.000399	0.002238	0.0005
0	0	0	5.18E-05	0	0	0	0	0
0	0.00646	0.011552	0.00845	0.014073	0.015497	0.012887	0.014834	0
0.000525	0.00058	0.001722	0.001129	0.001733	0.00276	0.003013	0.003213	0.003642
0.048981	0.090056	0.068027	0.064726	0.106789	0.097287	0.070221	0.045058	0.060015
0.014129	0.020252	0.019594	0.015561	0.017616	0.037337	0.026383	0.066075	0.037191
0.000488	0.001844	0.00182	0.000942	0.003084	0.009061	0.001489	0.001752	0.001407
0	0.000142	0.000158	0.000221	0.000263	0.000326	0.000136	0.000176	0.000139
0.001419	0.002368	0.003621	0.010432	0.004985	0.007202	0.005877	0.004839	0.006202
0.016656	0.016238	0.016308	0.01667	0.016915	0.01727	0.0181	0.017588	0.017118
0.020731	0.022134	0.021492	0.015078	0.114352	0.073679	0.072688	0.048982	0.027906
0.001647	0.002834	0.005	0.00555	0.008655	0.007916	0.005845	0.008207	0.006462
0.003881	0.012415	0.009594	0.00195	0.02428	0.007723	0.004976	0.011444	0.015832
0.000272	0.00028	4.42E-05	0.000256	0.001199	5.79E-05	0.001087	0.000554	0.000514
0.010706	0.029098	0.033776	0.013391	0.117278	0.057859	0.109768	0.113328	0.024175
0.017009	0.038026	0.024341	0.02412	0.053627	0.04368	0.046837	0.063929	0.034966
0.00358	0.003229	0.005753	0.004834	0.008439	0.00946	0.008436	0.007127	0.011248
0.00014	0.000383	0	0.002831	0.000153	0.001448	0.000251	0.000656	0.00019
0.048215	0.132093	0.103743	0.052836	0.130004	0.101595	0.122841	0.166492	0.102079
2.91E-05	3.27E-05	7.57E-05	8.05E-05	3.68E-05	9.4E-05	0.000194	0.000115	4.18E-05
0.003276	0.007956	0.006244	0.006383	0.013326	0.013235	0.011472	0.044831	0.016688
0	0	0	8.17E-05	0	0	0	0	0
0.02976	0.096216	0.095471	0.039853	0.111578	0.192058	0.033942	0.070502	0.063749
0.004567	0.009006	0.008211	0.019943	0.009473	0.021582	0.00607	0.008043	0.010707
0.000211	0.000672	0.001092	0.006419	0.000747	0.001544	0.00121	0.000896	0.000969
0.000115	0.000137	0.00017	0.000188	0.000288	0.000299	0.000409	0.000286	0.000294
0	0.000908	0	0	0	0	0	0	7.2E-05
0.3756	0.552374	0.54484	0.548252	0.692056	0.665548	0.565012	0.733622	0.676189
0.000413	0.0007	0.001433	0.002876	0.001986	0.001268	0.001085	0.000834	0.001032
0.000981	0.002424	0.003048	0.00998	0.004117	0.007388	0.004464	0.004099	0.00413
6.93E-05	0.000228	0.000159	0.001978	0.000516	0.000364	0.000502	0.000581	0.000408
0.001042	0.001054	0.002864	0.003227	0.002357	0.003502	0.001162	0.004459	0.001497
0.000202	0.000295	0.000407	0.000683	0.000772	0.001206	0.000837	0.000812	0.000462
0.000328	0.004091	0.000932	0.000683	0.000954	0.000837	0.006443	0.001431	0.000918
0	0.00031	0	0.011616	0.000418	0.005619	0	0.000421	0.000346
0.013579	0.036928	0.029704	0.017395	0.037621	0.039704	0.032814	0.042888	0.02882
1	1	1	1	1	1	1	1	1
0.000224	0.000285	0.000372	0.001417	0.000393	0.001053	0.000545	0.000439	0.000435
0.000809	0.001876	0.001535	0.000492	0.00195	0.001503	0.002135	0.002675	0.00176
0.000438	0.00054	0.000517	0.000862	0.000657	0.000729	0.001281	0.00073	0.000649
0.001571	0.002419	0.003738	0.010455	0.004985	0.007306	0.005914	0.004889	0.006384
0.004093	0.012886	0.006844	0.002479	0.007614	0.013965	0.00872	0.006687	0.006156
0	0	0	0	0	0.000359	0	0	0
0.004265	0.005888	0.007403	0.0151	0.008989	0.013024	0.009696	0.008973	0.009962
0.000155	0	0.000136	0.00101	0.000204	0.00086	0.000382	0.000286	0.000315
0.000496	0.001306	0.000896	0.000492	0.002532	0.000544	0.002706	0.002069	0.000752
0.000239	0.000445	0.000519	0.001984	0.000561	0.001117	0.001462	0.000833	0.000886

0.11768	0.247042	0.195253	0.086635	0.279791	0.348508	0.191403	0.168276	0.206591
0.000103	0.000253	0.000198	0.000656	0.000333	0.000348	0.000352	0.000351	0.000478
0.000121	0.000147	8.29E-05	0.000462	0.000214	0.000357	0.000369	0.000153	0.000244
0.013577	0.027413	0.026725	0.019306	0.138722	0.089969	0.045232	0.030255	0.03311
5.15E-05	6.45E-05	7.31E-05	0.000189	0.000221	0.000122	0.000169	0.000109	8.82E-05
0.004448	0.00452	0.006495	0.006538	0.010363	0.010884	0.007244	0.009671	0.007732
0.001196	0.002193	0.004627	0.003264	0.004256	0.003664	0.003117	0.008787	0.003808
0.000662	0.001062	0.001146	0.001231	0.001534	0.002881	0.002088	0.001599	0.001648
6.5E-05	0.000141	0.000284	0.001909	0.000558	0.000706	0.000488	0.000775	0.00069
0	0	0	0.000197	6.44E-05	4.57E-05	0.000157	8.26E-05	8.67E-05
0.019994	0.024748	0.024416	0.013862	0.135575	0.087181	0.071609	0.048318	0.026961
0.003996	0.005737	0.007549	0.007697	0.009799	0.009731	0.010469	0.009594	0.010043
0.001526	0.00157	0.001519	0.001041	0.008883	0.005701	0.005628	0.003808	0.002034
0	0	0	0	0	0	0	0	0
0.00016	0.000387	0	0.000316	0	5.09E-05	0	0	4.31E-05
0.000465	0.000794	0.00079	0.000909	0.001734	0.0018	0.001139	0.001334	0.001483
0	0.000238	0	0.000393	0	0	0.000335	0.000235	0.000198
0.00573	0.008642	0.018258	0.033479	0.013872	0.027634	0.024516	0.023446	0.033209
0.106444	0.272881	0.174913	0.217736	0.211411	0.384359	0.223201	0.347715	0.261667
0.000166	0.000432	0.000507	0.001595	0.000522	0.001682	0.000414	0.000549	0.001303
0	6.17E-05	7.42E-05	0.000152	0.000119	0.00016	0.000115	6.01E-05	0.000279
0.000521	0.000527	0.001432	0.001614	0.001178	0.001751	0.000763	0.002238	0.000997

CLL-12	CHL-1	CHL-2	CHL-3	CHL-4	CHL-5	CHL-6	CHL-7	CHL-8
0	0	0	0	0.000221	0	0	0	0
0.000996	0.000323	0.000223	0.000272	0.000434	0.000685	0.000466	0.000124	0.001033
0.007931	0.001492	0.000906	0.00036	0.002286	7.81E-05	0.001781	0.00092	4.68E-05
0	0	0	0	0	0	0	0	0
0.030243	0.01356	0.011092	0.012681	0.034371	0.01609	0.024331	0.008256	0.024861
0.001256	0.000713	0.000183	0.000176	0.003476	0.000998	0.001185	0.00011	0.001505
0	0	0	0	0	0	0	0	0
0.016503	0.003323	0.001119	0.00079	0.011184	0.004706	0.009816	0.001711	0.007689
0.002902	0	0	8.23E-05	0.000697	0.0006	0.001869	0.000705	0.001545
0.097953	0.064764	0.026133	0.04133	0.103749	0.046936	0.070206	0.025643	0.093177
0.039927	0.012033	0.021955	0.018176	0.027545	0.018658	0.020741	0.009117	0.020243
0.00528	0.000437	0	0	0.001801	0	0.001587	0	0.001117
0.000198	7.27E-05	0	0	0.000407	0	0.000113	2.6E-05	0.000199
0.005813	0.001983	0.000871	0.000708	0.016992	0.002559	0.00355	0.001664	0.002856
0.016434	0.015896	0.016348	0.016509	0.016343	0.016709	0.016195	0.017245	0.017052
0.116108	0.021967	0.014437	0.005492	0.034493	0.038355	0.027725	0.014288	0.054707
0.00631	0.003713	0.001704	0.001119	0.005448	0.003888	0.005127	0.001685	0.006999
0.011901	0.003842	0.002021	0.006656	0.007077	0.004955	0.008345	0.004708	0.006034
0.001125	0.000306	0.000134	6.14E-05	0.000531	4.02E-05	0.000343	0.000121	0.000641
0.055922	0.011693	0.024488	0.011196	0.095833	0.023904	0.050785	0.020244	0.079631
0.058475	0.018018	0.015457	0.02216	0.027448	0.03602	0.028763	0.014835	0.05321
0.010363	0.004401	0.000636	0.001224	0.012454	0.003179	0.004596	0.001549	0.009694
2.64E-05	0	0	0	0.004316	0.000168	0	0	0.00021
0.123558	0.052331	0.0459	0.048767	0.160699	0.068079	0.10275	0.035035	0.103234
6.15E-05	0.000255	0.00011	0.000138	0.000467	7.84E-05	0.000208	0.000101	0.000395
0.012361	0.004582	0.004712	0.010627	0.010392	0.005074	0.008128	0.010453	0.009994
0	0	0	0	0.000121	0	0	0	0
0.15411	0.047867	0.013944	0.031105	0.086306	0.044293	0.090456	0.017437	0.037129
0.012411	0.005554	0.003702	0.002417	0.031734	0.005722	0.009913	0.002547	0.006421
0.000396	0.000639	0.000435	0.000127	0.015654	0.0005	0.000197	4.02E-05	0.000466
0.000358	0.000163	0.000143	9.16E-05	0.000211	0.000133	0.000213	0.000121	0.000207
0	0	0.000489	0	0	0	0	0	0
0.727189	0.385058	0.157265	0.299535	0.573807	0.447304	0.479014	0.225247	0.609567
0.000811	0.000607	0.000316	0.000147	0.007747	0.000898	0.00051	0.000253	0.000761
0.006142	0.001452	0.000675	0.000389	0.018127	0.00217	0.003603	0.001042	0.002345
0.000287	0.000195	0	0.000148	0.001885	0.000108	0.000158	0	0.000415
0.002513	0.001426	0.000366	0.000351	0.006953	0.001995	0.002369	0.00022	0.00301
0.001167	0.00035	0.000133	0.000122	0.001271	0.000224	0.000293	0.000103	0.000809
0.002526	0.00027	0.000172	9.99E-05	0.030269	0.000459	0.000497	0.000373	0.000541
0.000302	0	0	0	0.01695	0.000249	0	0	0.000257
0.040008	0.015171	0.012336	0.013181	0.041587	0.01835	0.029597	0.00914	0.027027
1	1	1	1	1	1	1	1	1
0.000714	0.000264	0.00021	8.37E-05	0.002286	0.000256	0.000207	0.000176	0.000292
0.002128	0.00096	0.000623	0.000702	0.003041	0.000986	0.001508	0.000616	0.001522
0.00115	0.000293	0	0	0.001241	0.000445	0.000527	0	0.000977
0.006005	0.002203	0.000909	0.000793	0.017164	0.002551	0.003563	0.001621	0.002969
0.011204	0.003384	0.004123	0.003746	0.004133	0.004779	0.005757	0.001493	0.005985
0	0	0	0	0.000532	0	0	0	0
0.012458	0.005328	0.003313	0.003054	0.024532	0.005886	0.008368	0.004728	0.006543
0.000341	0.000155	3.69E-05	0	0.001143	0.000142	0.000148	5.8E-05	0.000147
0.001338	0.000518	0.000235	7.94E-05	0.000476	0.000401	0.000547	0.00018	0.001257
0.000596	0.000294	0.000135	0.000197	0.002956	0.000583	0.000537	9.42E-05	0.000564

0.300272	0.114496	0.058426	0.085583	0.13784	0.14009	0.145677	0.047003	0.144426
0.000321	0.000141	9.11E-05	0.000131	0.000777	0.00015	0.000308	5.13E-05	0.000201
0.000258	6.61E-05	5.01E-05	0	0.00056	0.000118	0.000135	0	0.000174
0.068432	0.014658	0.009244	0.004287	0.04198	0.024176	0.01838	0.017113	0.034728
0.000177	5E-05	0.000131	0.0002	0.000106	8.68E-05	7.62E-05	0.000139	0.000196
0.009212	0.008316	0.00377	0.002687	0.00692	0.008596	0.006292	0.003718	0.015832
0.002538	0.002845	0.000828	0.000343	0.009788	0.003776	0.003734	0.000413	0.003494
0.001453	0.000604	0.000246	0.00015	0.001559	0.000766	0.000462	0	0.001019
0.000447	0.000178	0.000162	0	0.00177	0.000234	0.000204	0	0.00034
6.64E-05	0	0	0	0.000186	0	6.39E-05	0	0
0.136399	0.025045	0.013983	0.005831	0.032867	0.044963	0.032428	0.01636	0.063207
0.011998	0.004052	0.002269	0.002791	0.010667	0.005167	0.007078	0.003707	0.006292
0.009112	0.001612	0.001113	0.00037	0.002446	0.002892	0.002072	0.001033	0.00408
0	0	0	0	0	0	0	0	0
0	0	0	0	0	0.000241	0	0	0
0.001791	0.000692	0.000239	0.000242	0.001738	0.000554	0.00088	0.00023	0.001267
0	0	0.000108	0	0.000535	0	0	0	0
0.020983	0.00461	0.003564	0.001601	0.036433	0.010908	0.010582	0.005824	0.01519
0.379851	0.122999	0.088977	0.197554	0.301005	0.206488	0.204097	0.098671	0.229838
0.00059	0.000212	0.000224	0.00019	0.002043	0.000514	0.000653	5.94E-05	0.00025
4.83E-05	0	0	0	0.000191	0	9.06E-05	6.13E-05	0.000158
0.001256	0.000713	0.000183	0.000176	0.003476	0.000998	0.001185	0.00011	0.001505

CHL-9	CHL-10	CHL-11	CHL-12	CHL-13	CHL-14	CHL-15
0	0	0	0	0	0	0
0.000596	0.00014	0.000356	0.000594	0.000278	4.99E-05	0.000982
0.005009	0.005103	0.002109	0.000546	0.000634	0.00109	0.010002
0	0	0	0	0	0	0
0.047476	0.053602	0.030199	0.023143	0.013779	0.026436	0.052905
0.001953	0.002434	0.000941	0.000684	0.000445	0.00033	0.001735
4.99E-05	7.34E-05	0	0	0	0	3.46E-05
0.018182	0.0204	0.00469	0.008182	0.013854	0.003618	0.018272
0.002382	0	0.00275	0.003026	0.002109	0.002518	0.003223
0.112783	0.000294	0.089055	0.064489	0.053861	0.105038	0.088704
0.008656	0	0.031338	0.010876	0.012887	0.034045	0.015807
0.004271	0	0.000469	0.004136	0.000651	0.000872	0.005344
0.000292	0	9.93E-05	0.000118	0.000114	0.000218	0.000335
0.006453	0.003613	0.005324	0.00619	0.003062	0.00461	0.005245
0.015815	0.000537	0.016715	0.01638	0.016839	0.016997	0.016786
0.073286	0.104138	0.03304	0.007758	0.009716	0.017296	0.144313
0.008118	0.013244	0.005614	0.003282	0.003005	0.006142	0.008074
0.032507	0	0.00501	0.002113	0.013529	0.008927	0.020941
0.000984	0.000169	7.1E-05	0.000223	0.000201	0.000315	9.56E-05
0.081702	0.05142	0.076088	0.061429	0.040225	0.076237	0.07881
0.045233	0.062814	0.023101	0.035552	0.019916	0.028137	0.05436
0.027735	0	0.003303	0.007434	0.004741	0.004703	0.014038
0.000619	0	0	0.000902	0	0	0.000524
0.188988	0.246385	0.133279	0.099738	0.058465	0.113971	0.19259
0.00011	5.59E-05	0.000313	0.000289	0.000197	3.47E-05	6.06E-05
0.021093	0.005121	0.025096	0.014777	0.01317	0.038509	0.01393
0	0	0	0	0	0	0
0.108569	0.001036	0.017511	0.099382	0.044963	0.039848	0.127384
0.011952	0.007523	0.005539	0.011145	0.005504	0.010918	0.010902
0.001161	0	0.000342	0.002019	0.000212	0.000766	0.001503
0.0003	7.83E-05	0.000252	0.000207	0.000197	0.000206	0.000338
0	0	0	0	0	0	0.001376
0.831311	0.933978	0.487907	0.527899	0.457268	0.616491	0.63569
0.000823	0.000698	0.00057	0.002245	0.000373	0.000743	0.001594
0.005053	0.002468	0.003373	0.005458	0.002827	0.002997	0.005718
0.000659	0	0.000285	0.0002	0	0	0.000426
0.003906	0.004868	0.001882	0.001369	0.00089	0.000912	0.00347
0.000877	0	0.000218	0.000448	0.000232	0.000377	0.001094
0.000714	0.001258	0.000275	0.000717	0.000442	0.00112	0.000709
0.002644	0	0.000252	0.003297	0	0	0.000346
0.052143	0.050457	0.031926	0.029267	0.017525	0.031353	0.056056
1	1	1	1	1	1	1
0.000543	0.000217	0.000415	0.000573	0.000209	0.00035	0.000504
0.003085	0.00045	0.001888	0.001541	0.000926	0.001825	0.002817
0.00143	0	0.001138	0	0.000507	0.000495	0.000876
0.006467	0.003675	0.005347	0.006163	0.003096	0.005195	0.005286
0.009607	0	0.004409	0.005758	0.00421	0.008917	0.013621
0	0	0	0	0	0	0
0.011358	0.003841	0.009487	0.009684	0.007162	0.007416	0.010676
0.00036	0	0.000293	0.000297	9.16E-05	0.000244	0.000194
0.001819	0.000543	0.00023	0.000942	0.000604	0.001229	0.001699
0.000995	0	0.000532	0.000769	0.000395	0.00079	0.000632

0.279329	0.206072	0.12016	0.144112	0.092455	0.166169	0.334317
0.000376	0	0.000171	0.000355	0.000201	0.000362	0.000467
0.000233	0	0.000108	0.000152	0.00015	0.000217	0.000152
0.044821	0.058482	0.021979	0.010057	0.006949	0.022143	0.085641
0.000232	0.000191	0.000104	5.27E-05	2.66E-05	4.62E-05	0.00052
0.009974	0.015266	0.006811	0.004292	0.007029	0.007366	0.009681
0.003591	0.008365	0.002773	0.002769	0.00145	0.003189	0.005234
0.002306	0	0.000808	0.001465	0.000399	0.001493	0.002768
0.000489	0.000308	0.000357	0.000631	0.00011	0.000324	0.000651
7.06E-05	0	3.7E-05	4.69E-05	0	5.31E-05	0
0.08258	0.104138	0.038107	0.008055	0.010311	0.015597	0.142936
0.011365	0.006462	0.006891	0.008546	0.006585	0.005823	0.012466
0.005544	0.00754	0.002536	0.000473	0.000669	0.001234	0.011476
0	0	0	0	0	0	0
0	6.5E-05	0	6.46E-05	0	0.000383	0
0.001603	0	0.001016	0.00081	0.000728	0.001436	0.001974
0.000331	0	0	0.000202	0	0.000168	0
0.019945	0	0.017283	0.021848	0.011084	0.021225	0.029725
0.492537	0	0.242183	0.286432	0.189715	0.245375	0.41036
0.000798	0	0.001286	0.000507	0.000228	0.000558	0.00041
0.000177	0.000178	4.67E-05	9.1E-05	6.64E-05	0.00011	6.94E-05
0.001953	0.002434	0.000941	0.000684	0.000445	0.000581	0.001735

LC-MS data

	Retention time	QC1	QC2	QC3	QC4	QC5
Cystine	1.774	0.025042	0.00478	0.013366	0.020917	0.029084
Asparagine	1.805	1.10403	0.313392	1.00633	1.39507	1.60375
Aspartic acid	1.793	0.257428	0.043717	0.074974	0.08869	0.229301
Serine	1.834	0.580383	0.176142	0.384609	0.469849	0.507658
Alanine	2.217	1.39922	0.090772	0.914933	0.996128	0.967762
4-Hydroxyproline	1.801	0.664391	0.414535	0.569484	0.601258	0.631803
Cystathionine	1.981	0.174314	0.229644	0.278661	0.2562	0.171123
Glycine	1.972	0.313787	0.160624	0.312811	0.31484	0.301904
Citicoline	1.848	0.023529	0.243158	0.090042	0.066794	0.028855
Glutamine	1.92	2.99782	3.22877	3.81877	3.24163	2.91561
Threonine	1.994	0.514038	0.227892	0.609783	0.560814	0.500165
Cysteine	1.999	0.00263	0.000717	0.001873	0.003023	0.002722
Dimethylglycine	2.591	0.171779	0.188074	0.126489	0.136517	0.134964
Methionine sulfoxide	4.086	0.078786	0.010326	0.053417	0.057455	0.063726
Glutamic acid	2.087	1.63214	0.280014	2.04195	1.87863	1.48183
Cytidine monophosphate	1.704	0.060981	0.164265	0.10005	0.076275	0.050719
Citrulline	2.141	0.24995	0.019879	0.249017	0.277279	0.24683
Guanosine monophosphat(	2.205	0.394831	0.313169	0.709312	0.686014	0.432444
Proline	2.24	11.1588	6.31608	7.99245	9.28931	9.95957
Ornitine	2.843	0.2129	0.083605	0.110824	0.128473	0.143779
2-Aminobutyric acid	2.59	0.034678	0.016202	0.046532	0.066133	0.068264
Histidine	2.97	0.551492	0.582738	0.728736	0.463128	0.533914
Arginine	3.527	0.518631	0.509895	0.743415	0.631785	0.494385
Creatine	3.26	151.324	25.1656	32.5166	142.541	147.646
Cytosine	4.145	0.074308	0.018322	0.032051	0.044657	0.060199
Choline	4.752	40.1079	17.9713	21.43	27.3486	33.4401
Valine	4.794	4.60186	0.794887	0.817766	1.28798	4.61483
Creatinine	5.241	97.0844	45.6087	54.1734	68.4108	81.6005
Norepinephrine	4.602	0.141549	0.382364	0.262427	0.22979	0.168017
Carnitine	5.727	3.32297	2.25045	2.66186	2.62869	2.63571
Methionine	5.348	0.348314	0.114937	0.111749	0.130978	0.364456
Niacinamide	5.231	2.16266	0.250102	0.803526	1.2836	3.30738
Thymine	5.958	0.011321	0.004623	0.004112	0.008258	0.009435
Histamine	6.308	0.990157	0.309089	0.294987	0.807639	0.901816
Guanosine	5.952	5.92796	4.15273	3.86232	4.71376	4.9043
Inosine	5.95	1.82425	1.31534	1.22846	1.5002	1.56487
FAD	5.976	0.000991	0.015678	0.003264	0.000496	0.000641
Pantothenic acid	5.983	0.888665	3.03883	1.77772	1.13509	0.857642
Cytidine	6.308	13.051	3.95451	9.48641	10.8109	11.8126
Adenine	6.409	0.115109	0.04381	0.021969	0.070088	0.088657
Tyrosine	6.552	2.99547	1.85075	1.78486	2.65985	0.084717
Adenosine	6.611	5.73518	0.459293	2.59165	3.50113	4.40223
Isoleucine	7.632	19.6794	7.20187	7.18946	7.9353	18.0788
Leucine	7.632	20.36	7.59084	7.54737	8.34104	18.8923
Phenylalanine	8.25	20.4509	6.03775	6.26384	7.92352	17.3413
S-Adenosylhomocysteine	8.682	0.095682	0.142075	0.109722	0.105228	0.080642
Kynurenine	8.642	0.052411	0.012628	0.015702	0.022094	0.042691
Acetylcarnitine	9.285	323.186	97.9409	133.517	187.437	241.656
Tryptophan	10.588	3.83638	0.969461	1.26974	2.61302	3.19809
Serotonin	11.719	0.007691	0.000193	0.002315	0.000398	0.009002
Allantoin	1.721	0.378224	0.037285	0.10943	0.19781	0.30166
2-Morpholinoethanesulf(	1.89	1	1	1	1	1

Methionine sulfone	4.219	0.04666	0.010567	0.053436	0.05344	0.044764
Malic acid	2.11	6.99348	13.1811	13.9945	12.3001	7.59574
Isocitric acid	2.235	2.70412	1.39218	1.35028	1.71411	1.8756
Lactic acid	2.243	0.280772	0.003195	0.015646	0.049122	0.246375
Guanine	5.548	0.00587	0.016579	0.009211	0.008322	0.006942

QC6	QC7	QC8	QC9	QC10	QC11	QC12	MHL-1	MHL-2
0.030384	0.029606	0.03074	0.027937	0.022121	0.008571	0.018222	4.02038	1.15902
0.754874	1.07626	1.03099	0.981719	0.674835	0.385125	1.01217	35.4828	14.7841
0.218056	0.233864	0.195752	0.080961	0.080485	0.056501	0.076445	44.839	21.9139
0.491308	0.515873	0.520755	0.500924	0.456431	0.244291	0.405782	15.03	7.39883
0.978032	1.04734	1.00076	1.25155	0.899198	0.229534	0.93146	76.2381	32.5123
0.515337	0.684997	0.616678	0.598768	0.499479	0.440662	0.513348	107.447	57.4395
0.13591	0.172537	0.154298	0.229293	0.182695	0.392839	0.286949	10.8473	2.63472
0.253918	0.28431	0.284688	0.282471	0.222002	0.237592	0.278578	9.7728	5.18548
0.020045	0.025019	0.02162	0.046747	0.026445	0.221205	0.077403	0.37221	0.18736
2.47204	2.94066	2.78067	3.14295	2.46791	4.10761	3.26189	407.115	231.078
0.463283	0.577026	0.52537	0.507998	0.4379	0.634415	0.540334	36.3564	29.4319
0	0.003941	0.005239	0.003095	0.001258	0.002055	0.001871	0.092364	0.060608
0.101366	0.146932	0.102652	0.175573	0.081036	0.028856	0.119355	0.925539	0.814859
0.047324	0.068077	0.065425	0.056468	0.041981	0.047568	0.05444	0.909604	0.353341
1.34295	1.47394	1.51306	1.7344	1.4468	1.02476	2.11665	451.271	165.284
0.059591	0.067361	0.065726	0.070994	0.056354	0.189152	0.089253	5.93313	2.1396
0.222918	0.241808	0.26404	0.285066	0.240668	0.041159	0.308173	6.43	4.14512
0.338329	0.376706	0.288405	0.433582	0.343373	0.387734	0.548851	2.45752	1.25763
10.0111	10.364	10.2032	9.42822	8.79037	5.73432	7.82526	142.261	69.3741
0.202396	0.16023	0.18898	0.169133	0.137338	0.112593	0.150024	4.90542	1.62983
0.063205	0.072829	0.065034	0.057349	0.0514	0.018334	0.050338	0.693317	0.576951
0.499176	0.588128	0.528714	0.582587	0.485146	0.642318	0.466411	50.7158	28.0906
0.421724	0.503695	0.449255	0.617012	0.455812	0.551243	0.704969	49.6827	16.5661
129.546	155.601	143.504	149.112	123.464	29.7274	27.938	851.9	336.148
0.054064	0.056746	0.060257	0.04658	0.04601	0.019099	0.037185	0.086468	0.311228
33.4436	35.8887	35.8757	28.8707	27.0801	16.2083	20.8425	64.8953	31.2172
4.1903	4.96893	4.60517	4.65705	3.98765	0.703664	1.03628	30.4116	17.9225
81.5399	88.2473	87.8427	71.9485	65.8617	41.1145	51.6798	60.4597	21.0503
0.04805	0.149363	0.188082	0.196066	0.08219	0.396705	0.245163	2.72974	0.914021
2.45285	2.90203	2.91246	2.92938	2.17586	2.67369	2.58904	54.6596	41.0519
0.275708	0.381806	0.355489	0.10649	0.298714	0.098955	0.101473	10.2657	4.01141
6.14323	1.62881	1.69428	1.35143	1.33051	0.517012	0.917569	19.3395	39.8838
0.012233	0.009422	0.012925	0.008414	0.011097	0.003087	0.006344	0.075928	0.051287
0.867247	0.942996	0.894307	0.835962	0.750763	0.290368	0.683629	4.1113	2.39672
4.45505	4.93432	4.78643	4.21238	4.16636	3.56236	3.47848	36.6833	3.87187
1.48674	1.6008	1.53625	1.29658	1.21668	1.10595	1.08342	6.27847	2.44363
0.001721	0.001023	0.001563	0	0	0.008744	0.001284	0.055816	0.032339
0.753765	0.891985	0.834235	0.858419	0.806898	2.30779	0.984798	60.9659	24.1816
11.0878	12.4505	11.9578	11.0239	9.83613	3.75672	8.79242	54.1244	31.6218
0.098559	0.087969	0.106201	0.078081	0.082009	0.034063	0.048257	0.070818	0.033992
0.119376	0.098414	0.120047	2.67201	0.070424	1.57665	2.18564	10.5155	5.43897
5.24302	4.90938	4.92427	4.31136	4.36073	0.414	2.77863	13.7197	6.39176
17.247	18.8813	17.7853	17.7471	15.6944	6.06311	6.49367	13.73	6.00987
18.1369	19.5934	18.4689	18.1856	16.3545	6.31976	6.17092	14.3667	6.30669
16.5629	18.2905	17.2831	16.1111	14.5988	5.10566	5.88516	30.5661	12.5588
0.169163	0.072178	0.178802	0.089736	0.184111	0.117998	0.095942	0.066951	0.018056
0.048473	0.051186	0.048888	0.037482	0.039716	0.011514	0.015407	0.025603	0.011804
304.951	273.979	276.038	226.838	232.664	93.7423	140.2	71.2036	32.7651
3.15495	2.59445	3.54385	2.91604	2.96839	0.986821	2.10769	9.86364	4.68388
0.00538	0.004619	0.000856	0.008481	0.008367	0.0033	0.000454	0.000863	0.000268
0.371695	0.337557	0.340373	0.216575	0.266659	0.04673	0.129321	0.285526	0.097781
1	1	1	1	1	1	1	1	1

0.048674	0.041602	0.041763	0.046683	0.04217	0.010234	0.053182	0.004118	0.002176
6.30081	7.98334	6.88902	11.2293	8.4535	13.4981	12.606	0.408974	0.391314
2.73592	2.46768	2.37625	1.67438	1.76227	0.995137	1.25871	9.93926	5.57267
0.276738	0.274044	0.247899	0.208257	0.220724	0.003627	0.033517	2.7552	1.4128
0.006473	0.007789	0.006655	0.007726	0.006601	0.013432	0.007852	0.023911	0.02757

MHL-3	MHL-4	MHL-5	MHL-6	MHL-7	MHL-8	MHL-9	MHL-10	MHL-11
0.99066	2.07043	1.14351	1.63064	1.84796	2.32018	1.13122	0.676515	0.749748
31.8193	24.5443	13.4341	17.5049	24.238	9.02443	18.8676	17.5578	20.0421
21.6489	32.4493	22.339	22.8123	53.6506	9.94412	25.8931	19.2146	21.8895
14.2415	8.37455	7.20075	11.8276	6.61648	5.48997	6.61773	5.90442	8.39772
78.5585	39.0061	31.7386	49.9695	49.0433	18.7396	45.1221	25.9369	39.1901
76.171	71.4082	46.5002	44.4211	48.9404	55.529	65.0049	58.3049	46.3243
1.37068	1.22523	4.55926	3.6615	1.56704	7.27896	0.727298	3.83816	5.41722
10.3743	7.57874	2.99241	5.90747	5.68493	2.50155	5.95874	4.66322	3.41277
0.170268	0.250791	0.384304	0.098246	0.206251	0.639746	0.091455	0.128204	0.101249
332.209	247.486	164.028	264.035	206.954	123.888	270.854	168.058	272.404
11.535	31.6835	27.8176	34.6866	19.7118	18.6053	20.2386	20.1739	30.1036
0.015899	0.066095	0.056735	0.070487	0.054963	0.032257	0.041603	0.026234	0.058458
1.54959	0.476261	0.492784	1.98935	1.95378	0.567004	1.25786	0.567995	0.782243
1.14265	0.441029	0.302163	0.456722	0.487115	0.322238	0.440932	0.343441	0.287629
251.388	237.733	115.339	177.272	259.308	91.4492	177.68	126.003	132.813
0.702677	0.408267	1.79762	0.867568	2.15198	3.13155	1.67824	2.23929	0.754597
7.24827	19.4202	16.3046	3.75074	20.2408	7.87923	4.93921	11.6619	12.4665
3.12721	1.88696	1.16062	0.323991	1.42877	2.6542	1.42931	2.70724	1.38199
182.27	126.992	62.4201	111.24	110.361	35.7237	120.507	92.2504	95.9413
4.00717	2.76108	1.43357	1.15655	2.25658	2.04695	1.85149	1.73255	2.21379
1.64856	0.328484	0.342697	1.41388	1.08388	0.397809	0.798568	0.274534	0.369717
51.461	38.0036	22.7373	46.736	25.6212	19.1583	32.4554	19.5946	33.1826
44.4263	35.6559	19.9924	18.5818	15.8668	30.3255	28.417	23.7527	23.8183
613.665	580.964	332.115	334.517	500.089	242.292	524.703	477.463	461.177
0.620021	0.046918	0.157877	0.369594	0.971092	0.023689	0.093379	0.115701	0.180996
130.588	71.4678	15.4421	31.1673	46.8228	13.3405	37.1757	37.1399	38.4997
48.0835	15.5028	16.4515	29.5115	26.8673	2.61829	24.1836	14.0936	16.3252
95.9897	30.9219	50.5936	62.8055	94.0712	29.1925	91.8787	196.233	52.1681
1.60601	1.77038	1.45695	0.74037	2.1151	2.48581	0.807829	1.31124	1.36543
106.59	24.9671	38.7933	58.4182	47.8338	42.1026	96.5392	47.3588	54.6392
24.4881	4.77638	3.92028	6.86866	7.12321	0.802917	6.93039	4.54607	6.1141
87.4606	14.4805	2.49522	9.85227	10.4993	1.40939	5.45186	22.0548	23.1003
0.07126	0.069466	0.006348	0.025091	0.099643	0.004014	0.020316	0.036462	0.062836
23.9487	5.26789	1.92267	3.14908	2.5945	1.5142	3.79684	3.36564	2.46017
125.906	114.514	3.47402	2.29423	34.0079	2.91294	5.46166	7.78476	8.05949
5.8829	5.75391	2.52894	4.85883	6.65855	2.50462	5.50009	3.56807	3.02273
0.033429	0.053185	0.037356	0.024698	0.045486	0.093686	0.0128	0.030517	0.027221
50.414	40.379	19.87	30.0748	49.2075	16.5809	22.5026	37.6627	40.5215
292.291	66.216	25.4103	41.2054	31.5435	19.8258	51.1529	43.6033	33.1513
0.325769	0.05831	0.022252	0.045274	0.062135	0.00694	0.039837	0.077019	0.04077
20.6238	7.92656	4.9576	9.02836	6.76522	2.75795	11.1596	6.04611	8.47313
1.77421	3.04447	3.63683	9.57864	13.264	3.16948	15.3618	11.1659	8.72282
25.973	9.71623	4.32833	9.05527	11.3706	1.64453	8.35	5.90826	6.37262
27.5134	10.0949	4.48926	9.53225	11.8765	1.69339	8.73691	6.22964	6.74048
73.4104	24.8287	8.24353	21.8007	20.3145	3.42369	21.9149	14.2646	18.0211
0.009259	0.020644	0.009857	0.017681	0.044537	0.009077	0.014973	0.02353	0.026229
0.044753	0.005742	0.005211	0.016926	0.020372	0.001325	0.011861	0.004095	0.011355
89.5208	33.4738	25.5617	44.4618	60.9987	14.7745	59.4195	47.9579	66.6109
30.1123	9.33975	3.04105	6.86029	6.46676	1.52633	7.11737	5.36814	7.46211
0.002256	0.000782	0.000197	0.000233	0.000903	0.000205	0.000738	0.000213	0.000615
0.199176	0.145646	0.058842	0.115256	0.21884	0.024616	0.204812	0.190441	0.164127
1	1	1	1	1	1	1	1	1

0.004285	0.00141	0.000854	0.003023	0.002553	0.000653	0.002301	0.000959	0.0009
0.109499	0.134243	1.42178	0.230648	0.51232	1.12937	0.378009	0.321016	0.186731
2.678	3.77999	11.5832	5.75615	7.68766	11.2623	4.28963	7.79447	6.93918
3.15357	0.08922	0.845955	1.0629	3.54693	0.305123	1.34057	1.37743	1.72803
0.159061	0.044895	0.020778	0.013837	0.024867	0.001807	0.050566	0.047267	0.044782

MHL-12	MHL-13	MHL-14	MHL-15	MLL-1	MLL-2	MLL-3	MLL-4	MLL-5
1.00692	1.72515	1.20261	0.911852	1.2114	1.06753	1.58363	1.09857	2.55346
16.4778	21.72	16.8996	20.2684	24.2375	22.165	21.6665	9.77196	15.8357
23.3004	12.836	17.2094	15.48	7.86693	24.7735	18.4682	23.0127	9.9007
5.34649	8.5383	9.07543	9.19821	9.49343	13.0538	8.12713	6.13941	13.0828
27.968	34.9069	50.6455	35.224	44.0615	48.9785	33.1762	19.6267	34.652
43.5622	39.7249	72.1327	83.5856	84.0986	70.0468	63.696	26.0981	27.504
1.19079	2.21996	1.0986	1.08778	4.68198	3.04674	7.5955	1.22784	5.42319
4.16336	6.46607	6.46356	5.61074	10.1744	6.62147	4.43495	2.8519	4.1391
0.357688	0.132797	0.138713	0.161847	0.080339	0.137368	0.103803	0.179141	0.160845
180.718	308.728	260.711	351.729	336.524	243.408	198.514	126.59	199.278
22.9554	28.2706	24.9013	32.6707	30.4591	40.8388	28.0245	8.83217	41.7663
0.053812	0.053654	0.044095	0.079799	0.082203	0.072246	0.095319	0.016976	0.079064
0.68065	0.984564	1.5622	0.806251	1.44896	1.4766	0.785056	0.38784	1.15067
0.42052	0.390388	0.443451	0.407412	1.2458	0.504377	0.451841	0.17755	0.550571
148.246	116.298	127.857	150.197	102.69	151.099	118.794	135.372	94.1564
2.80404	0.261113	1.35642	2.4416	0.269049	2.03193	3.47974	1.55437	3.79186
15.3818	3.0423	9.35327	9.75484	11.9775	7.97995	5.14843	3.81151	7.03499
2.42171	1.18842	1.45402	3.3907	0.310753	0.875865	1.56648	1.21039	2.69239
81.0534	158.47	91.2366	123.515	184.969	83.4245	110.364	97.5673	109.493
1.40094	1.42629	1.6509	2.41723	3.06368	1.61156	1.86144	1.65542	1.38343
0.363464	0.868872	1.15524	0.606282	1.04378	1.02523	0.419804	0.23469	0.824225
20.1801	39.2571	25.4242	36.3026	45.3663	31.1354	27.0693	21.7151	35.8907
13.1854	31.9004	20.9111	26.13	37.9268	24.8374	29.8976	31.0827	30.1333
456.259	581.771	440.839	670.065	671.756	453.955	569.484	466.168	343.936
0.111847	0.536773	0.760347	0.177488	0.213045	0.912296	0.067484	0.345606	0.564037
34.6857	76.0355	44.4896	52.0932	358.14	51.0069	37.6344	60.7089	67.4441
19.0101	32.1602	23.9119	19.1538	45.0582	29.4131	23.0858	20.479	47.3121
43.7069	130.648	176.012	130.024	107.614	26.5522	57.7892	35.9543	77.3084
1.55219	1.38065	0.712192	1.04263	1.0179	1.22843	1.56205	1.56951	1.60786
45.5781	49.21	31.8173	53.1349	48.4647	41.6741	72.6816	43.5602	40.5521
3.47205	7.47107	6.23433	8.32641	18.0425	8.59467	7.12645	2.4052	12.8367
11.1638	44.5223	5.92833	19.0979	170.827	8.12092	45.6624	19.5172	26.5287
0.057019	0.087737	0.015105	0.077341	0.042179	0.021082	0.070587	0.018715	0.014031
2.20325	2.64096	3.29946	4.64591	6.92183	4.85272	3.80911	2.67505	6.74095
4.09159	11.5946	4.20321	7.42285	13.7711	4.69538	4.13082	4.26847	5.52364
6.48662	6.23989	3.46357	5.33088	4.90524	5.36412	5.1625	2.87703	2.31382
0.044433	0.022524	0.017001	0.02984	0.013233	0.026347	0.033961	0.031705	0.030593
42.6223	44.4218	20.6607	55.3549	48.5632	18.064	38.9542	18.7944	21.7591
30.0869	34.9284	43.1405	60.0638	92.3978	63.8774	51.574	35.6002	85.4008
0.099714	0.04633	0.070491	0.16203	0.05662	0.05864	0.049442	0.026013	0.115269
4.52024	10.0358	7.27406	9.63073	16.0297	9.50251	8.37047	5.71685	11.4918
17.0668	8.78779	8.46419	10.0279	10.2476	10.2361	13.1415	8.1304	4.00901
5.43874	11.2383	6.19004	11.296	23.0368	8.3545	10.2111	7.59181	10.6631
5.73965	11.7037	6.47102	11.9612	24.2226	8.82921	10.764	7.97055	11.1468
11.7985	27.5425	15.098	30.7219	46.2464	20.6878	25.7686	17.5112	26.0854
0.018107	0.030087	0.006086	0.012354	0.035135	0.013672	0.029742	0.027283	0.01651
0.007557	0.024534	0.010343	0.014957	0.648364	0.012286	0.016014	0.005078	0.050441
47.7564	50.3114	31.0245	50.2313	78.052	42.1536	78.4955	37.718	36.9421
3.98441	11.7356	5.67857	10.3753	18.9196	7.98746	9.49572	5.68411	10.835
0.00036	0.000332	0.000218	0.000613	0.000832	0.000208	0.00045	0.000231	0.000887
0.155429	0.164759	0.096798	0.161896	0.220199	0.11557	0.202103	0.069226	0.132057
1	1	1	1	1	1	1	1	1

0.001328	0.001867	0.001984	0.001782	0.002951	0.001038	0.001795	0.001805	0.002882
0.620494	0.111722	0.378217	0.155691	0.029009	0.702981	0.291991	0.447479	0.659562
7.79112	5.59103	5.69655	4.00357	1.06397	11.6585	7.59684	10.2157	14.1289
1.46112	1.58295	1.29466	2.26724	4.77572	1.00269	1.754	1.87799	1.21222
0.022158	0.041807	0.034332	0.04823	0.110054	0.036347	0.031137	0.004098	0.075998

MLL-6	MLL-7	MLL-8	MLL-9	MLL-10	MLL-11	MLL-12	MLL-13	MLL-14
1.34414	1.5806	1.29877	1.45575	2.22091	2.20707	0.83388	0.911929	1.79423
10.8129	9.14175	10.6157	13.0919	33.9529	24.9389	11.8098	11.3441	15.7036
10.1523	23.7731	10.1393	14.3482	17.6711	6.39096	22.0721	23.0208	19.0868
11.1382	6.17236	5.57973	9.74021	17.5277	6.49383	6.2849	7.68687	9.25811
25.0975	21.6229	13.3671	28.4315	48.1367	16.3112	35.1592	24.5009	28.8342
13.8301	45.1456	14.2508	31.374	50.4689	29.5629	21.0909	20.9184	51.2608
3.16777	4.26374	1.54714	5.23114	5.32962	2.43556	1.57723	1.02989	4.7451
3.28816	3.74538	2.0573	4.1464	6.48318	2.59085	2.74077	2.54021	6.07449
0.339603	0.170308	0.273072	0.45784	0.195827	0.103649	0.097147	0.289805	0.458587
147.75	177.238	110.244	219.001	310.774	87.7593	238.97	142.289	223.976
25.5507	19.7286	8.69141	19.6981	27.6713	7.3147	9.66783	20.0756	43.2353
0.048359	0.040405	0.016851	0.042129	0.038881	0.037803	0.027225	0.045958	0.075802
0.455284	0.523277	0.219693	0.542562	0.758554	0.294049	1.14374	0.364999	0.456193
0.435796	0.324652	0.207405	0.349412	0.67197	0.207956	0.398907	0.257402	0.462932
103.992	137.044	79.7163	169.221	149.895	56.8208	114.138	122.94	154.103
1.9741	2.31999	0.655796	3.12416	2.57263	0.085935	0.221812	0.962177	2.87819
10.2276	7.76529	5.59373	12.3491	14.4344	1.92485	3.2951	4.55617	6.02172
0.813931	1.50062	0.434864	1.92027	1.60642	0.203261	0.073298	1.43615	2.50213
93.8066	63.3441	59.0363	106.089	173.773	135.985	95.3313	95.8414	72.656
1.83924	1.72088	1.41984	2.43637	4.70091	2.2892	1.39149	1.19698	2.38059
0.201206	0.349009	0.150963	0.286051	0.767288	0.129866	0.964535	0.259527	0.309416
35.8069	22.275	17.4647	31.7944	61.0873	19.5968	30.8799	24.5506	26.3501
46.6432	20.1264	21.4834	44.3048	42.4663	38.9202	29.9349	25.523	22.6047
69.4386	321.735	311.473	346.989	797.712	817.581	354.289	417.437	321.63
0.39253	0.314863	0.311348	0.187759	0.108071	0.058627	0.149457	0.140658	0.175423
62.4487	19.7389	53.0823	43.5713	153.654	164.023	38.53	36.8528	25.969
7.99808	18.6084	7.92563	22.9624	51.1783	11.1221	29.4467	25.4518	23.5815
50.893	41.7059	22.5005	42.5985	83.9473	92.3043	84.6248	55.2675	86.3026
0.181327	1.24865	1.23398	2.88234	0.339822	2.77082	1.00515	1.15678	1.73323
25.2203	26.3384	26.4306	34.2584	60.8805	51.1116	72.1012	19.0219	35.5152
3.14476	8.87917	3.38326	7.13912	14.2041	6.19567	6.25595	7.51868	3.38373
7.16394	5.61392	11.5986	7.22512	95.2944	40.0045	12.2938	3.97212	15.1201
0.011779	0.008409	0.013542	0.057601	0.074917	0.123632	0.009175	0.012823	0.026082
5.20329	2.50616	2.51336	5.09204	4.79815	5.95013	3.64039	2.19048	1.85696
7.64031	5.70687	7.49399	8.67051	43.2885	26.9887	13.3632	7.03877	3.67218
2.23548	3.09861	1.275	3.06217	6.00809	4.01883	2.35988	1.70756	2.73946
0.017467	0.022242	0.032232	0.068253	0.01415	0.005681	0.006244	0.028344	0.047709
13.875	18.6607	15.6399	32.7567	28.3277	46.1347	9.67278	21.4059	20.9234
67.4056	33.1372	33.2278	65.6874	64.899	79.8944	48.0399	28.9135	23.8097
0.07197	0.046507	0.020404	0.053999	0.062339	0.060942	0.030892	0.033545	0.030398
7.81349	4.04783	3.38176	6.29778	12.1467	0.064328	6.10311	6.48324	3.62931
2.20956	7.79583	2.3947	3.08236	19.3536	4.33121	2.19112	2.95755	7.78603
7.07188	3.84837	3.2276	6.21842	21.8385	6.48962	6.43617	6.21731	5.15286
7.40186	4.03953	3.33647	6.52633	23.1104	6.65466	6.63298	6.51203	5.41456
18.4036	8.10531	7.73374	14.0467	39.7768	19.5138	13.4864	14.9301	8.94557
0.003088	0.011266	0.022045	0.0168	0.074094	0.057647	0.011783	0.017405	0.011781
0.00567	0.005726	0.001594	0.005568	0.024569	0.004544	0.017984	0.005243	0.006935
26.9218	18.8852	33.3975	38.8934	85.9137	111.452	33.6072	23.3726	17.4411
7.43449	3.21754	2.73596	6.44069	12.5119	9.45181	3.98008	6.72991	2.80767
0.000281	0.000218	0.000157	0.000182	0.001135	0	0.000892	0.000279	0.000105
0.057828	0.082375	0.062031	0.071488	0.331749	0.23437	0.050007	0.086018	0.03817
1	1	1	1	1	1	1	1	1

0.00035	0.00097	0.001431	0.000919	0.001734	0.000921	0.001156	0.000502	0.002645
0.462016	0.606959	0.127005	0.738014	0.087233	0.062704	0.152872	1.56967	0.543801
13.903	8.41553	7.73728	18.7172	1.31373	2.06862	5.04091	7.64671	10.3014
1.51647	0.761812	1.41422	1.48264	0.094308	1.99359	2.11514	1.45722	1.32402
0.006445	0.02912	0.032954	0.035941	0.060143	0.056654	0.039652	0.056907	0.016216

MLL-15	CLL-1	CLL-2	CLL-3	CLL-4	CLL-5	CLL-6	CLL-7	CLL-8
1.08746	0	0.000323	0.006774	0.003978	0.001091	0.040806	0.00624	0.000623
20.032	0.944279	0.070553	0.553213	0.377016	0.437891	0.644008	0.205549	0.357336
18.6377	0.098346	0.008732	0.185739	0.05517	0.051233	0.353883	0.020954	0.011549
7.78695	0.677415	0.052542	0.782733	0.189713	0.14874	1.32133	0.21231	0.105152
21.5578	1.0229	0.060332	0.569996	0.104395	1.04273	2.78736	0.38914	0.294617
19.9209	0.67627	0.093408	0.454545	1.97093	0.596963	0.453371	0.157333	0.221787
2.85365	0.116852	0.011676	0.109036	0.871178	0.03649	0.032155	0.014552	0.030029
3.72771	0.081132	0.011508	0.41496	0.10017	0.093818	1.1546	0.07063	0.051358
0.378372	0.3167	0.35659	0.217866	0.519942	0.011814	0.037717	0.003908	0.093411
134.552	1.6749	1.21826	2.00293	3.89468	2.51733	4.77657	1.09623	1.23949
12.9152	0.441627	0.141847	0.460827	0.380115	0.411443	1.09492	0.222451	0.175508
0.030934	0	0.00036	0	0.00079	0.002299	0.001722	0	0.000673
0.380342	9.51164	0.122842	2.33039	0.067027	0.070697	1.48313	0.022907	0.032285
0.28073	0	0.005056	0.032881	0.006353	0.036274	0.176838	0.023833	0.007686
123.439	1.95713	0.120759	0.85555	0.375228	0.748781	2.7996	0.276116	0.210006
2.00141	0.292749	0.018902	0.063471	0.065917	0.002591	0.012373	0.002095	0.06032
7.86084	0.262714	0.006403	0.089489	0.028331	0.124709	0.09161	0.203456	0.054702
2.473	15.9666	1.06776	12.8188	1.39326	0.738902	2.35071	0.072431	0.70724
104.828	87.9491	3.23388	48.7396	8.62602	3.75871	139.102	4.02747	3.42992
2.24808	3.07052	0.047903	0.870682	0.221241	0.068011	3.37889	0.109578	0.064954
0.178219	2.64054	0.028903	0	0.045248	0.027371	0.688726	0.006578	0.013733
30.8865	3.82224	0.123589	3.7527	1.09132	0.192916	18.7111	0.28294	0.234622
42.4343	11.4754	0.132205	4.917	1.37668	0.107648	17.9557	0.024575	0.185288
403.019	459.873	18.6031	269.362	59.2316	9.31005	216.92	47.463	17.8572
0.155082	0.906666	0.016729	0.287909	0.049893	0.061149	0	0.007689	0.04057
100.806	57.9266	7.46467	61.0775	8.40234	5.20227	534.549	8.2878	5.04321
18.495	3.09866	0.18151	4.68648	0.79304	1.42237	19.9572	2.00464	0.208843
58.8128	339.755	26.4354	344.468	59.5829	21.4969	149.22	21.1084	22.0616
1.94379	14.1709	0.18303	3.73536	0.63626	0.019493	1.87119	0.0092	0.005144
20.379	39.709	1.34979	30.3826	7.91045	1.97528	23.6885	0.991166	2.06379
7.41221	0.505029	0.022995	0.781857	0.053419	0.018605	2.71796	0.101169	0.021186
17.802	2.67535	0.076848	0.610226	0.245054	0.265521	3.94793	0.734858	0.103527
0.055021	0.378425	0.00095	0.064605	0.016798	0.002972	0.018622	0.00237	0.005023
4.67058	5.09451	0.220253	2.12013	0.283816	0.293284	4.39507	0.145508	0.178366
7.315	57.6199	1.87956	49.9614	5.91255	1.07479	81.7402	1.08062	1.58732
2.58904	23.8544	0.802005	15.8825	3.60623	0.845441	14.3683	0.446113	0.455222
0.041553	1.5739	0.016648	0.123726	0.101814	0.001123	0.129115	0.000184	0.004037
25.4413	84.8785	1.62033	41.7389	6.20228	0.895934	31.6159	0.308886	2.85543
62.8827	64.7575	2.85953	26.9273	3.60249	3.80208	53.1034	1.9601	2.21684
0.044809	0.22993	0.004382	0.172523	0.046587	0.022731	0.096232	0.008937	0.0149
6.60164	8.3618	0.496323	10.7047	1.26006	0.764073	73.0303	0.120118	0.58763
6.57251	6.56118	0.161786	2.43728	0.821858	0.901527	3.11299	0.273834	0.780444
8.69965	24.8867	1.59895	40.4102	3.16687	1.51576	184.722	5.26575	1.37203
8.94783	25.734	1.69134	41.7021	3.27089	1.64743	192.779	5.54044	1.42925
18.5831	41.9218	1.49757	33.2089	4.51827	1.61385	153.792	3.19015	1.52082
0.031715	2.25725	0.085364	1.00723	0.198311	0.051266	1.10919	0.057885	0.053229
0.004372	0.010461	0.000398	0.056727	0.004226	0.003589	0.954018	0.006415	0.001527
34.2525	1415.55	56.6634	1068.54	132.998	74.1572	1304.52	102.726	63.2542
8.89907	5.34526	0.208509	6.43722	0.980118	0.345504	43.6351	0.616499	0.291222
0.000477	0.064698	0.000998	0.001933	0.004097	0.003969	0.002398	0.000856	0.000161
0.127933	0.331564	0.026615	0.381816	0.042194	0.056022	0.432759	0.084174	0.039907
1	1	1	1	1	1	1	1	1

0.000729	0.150943	0.009817	0.13003	0.018075	0.064962	0.131482	0.035375	0.021866
0.425593	207.278	8.12465	143.774	21.1797	4.71336	116.645	0.883061	5.56029
9.38302	11.1066	0.444142	17.7062	1.211	0.736366	45.2281	0.87003	1.09125
3.84226	0.402489	0.019033	0.218048	0.032894	0.014137	1.0226	0.055637	0.007642
0.050273	0.778405	0.012189	0.194036	0.036651	0.011406	0.087115	0.001101	0.008906

CLL-9	CLL-10	CLL-11	CLL-12	CLL-13	CLL-14	CLL-15	CHL-1	CHL-2
0.001691	0.033588	0.002276	0.000772	0.006706	0.004579	0.002332	0.021361	0.000721
0.314283	0.592957	0.210415	0.192946	0.487033	0.946612	0.378096	0.771372	0.065524
0.058783	0.226807	0.034515	0.018429	0.067823	0.095371	0.035701	0.103658	0.018933
0.160726	0.972914	0.141288	0.097878	0.376454	0.608465	0.144914	0.756077	0.065551
0.089871	0.205713	0.197175	0.103305	0.386532	0.75696	0.275659	2.77775	0.367435
0.207226	0.841379	0.126391	0.170546	0.46705	1.15901	0.238278	2.8846	0.28689
0.090775	0.175908	0.227929	0.078661	0.896305	0.796914	0.338744	2.53961	0.049965
0.103761	1.57656	0.041674	0.02132	0.08617	0.149584	0.074319	0.34627	0.027185
0.984812	0.111511	0.262641	0.357455	0.363584	0.09443	0.193304	0.174919	0.156978
3.79261	4.87811	1.68003	2.54027	4.10499	2.55562	2.53697	4.60492	1.9847
0.165187	1.20367	0.179612	0.101913	0.631546	0.678515	0.285529	1.43159	0.417289
0.000761	0.001957	0.001508	0.000335	0.001455	0.000668	0.001219	0.002281	0.000987
0.667149	28.9051	0.06992	0.096879	0.045949	1.76894	0.079742	2.93844	0.076949
0.00916	0.142322	0.013732	0.004548	0.019143	0.017175	0.008453	0.036368	0.022101
0.267556	4.61934	0.470941	0.069197	0.613968	1.51006	0.343698	2.85985	0.757466
0.035446	0.020331	0.032525	0.011891	0.139025	0.049436	0.313669	0.258997	0.200254
0.002878	0.286285	0.018168	0.009041	0.118047	0.141447	0.021311	0.290135	0.023879
1.87721	3.92179	2.39349	0.434111	4.19755	6.43056	2.9587	9.46531	0.368011
8.18869	144.601	3.68066	6.22966	4.91216	72.1829	3.55236	66.4249	3.58921
0.255981	2.72033	0.105347	0.074746	0.255186	1.09321	0.122063	1.85393	0.038055
0.21579	2.01318	0.017244	0.012539	0.028729	2.41099	0.018547	0.587856	0.050614
0.82491	12.5876	0.348896	0.36813	0.67943	4.76104	0.223805	9.0122	0.296705
0.740341	15.8351	0.591178	0.209953	1.02103	11.8159	0.631568	14.3136	0.191027
41.7182	474.544	25.698	26.0036	31.2716	432.267	22.7957	441.826	20.6264
0.057963	0.312022	0.036937	0	0.048937	0.204955	0.047425	0.176689	0
24.0205	222.179	8.5285	15.8374	10.4592	119.43	9.08104	75.8091	6.20314
1.51151	22.3195	0.240077	0.731383	0.25205	5.20119	0.278092	14.0597	0.30938
64.8275	290.243	27.9241	39.4334	41.9922	182.914	31.9454	73.0359	15.934
0.708783	5.16106	0.471858	0.147945	0.69852	3.09002	0.343962	7.51873	0.104019
2.7663	51.3437	3.26989	3.37747	1.87223	25.3676	2.58009	50.4318	2.87594
0.162231	2.64523	0.031105	0.062941	0.053476	1.06274	0.028974	1.8197	0.04454
0.202399	3.99588	0.495435	0.105648	0.720589	0.429423	0.686558	1.2322	0.092211
0.015002	0.135456	0.003417	0.002318	0.004372	0.090626	0.004737	0.076011	0.004872
0.47669	2.89027	0.156343	0.209018	0.341736	3.35027	0.342624	4.80951	0.164256
8.74988	92.1328	1.70581	2.98555	3.71802	65.1658	1.765	66.09	1.17278
3.17344	20.9042	0.634972	0.73461	1.00881	20.3632	0.923061	20.4725	0.8013
0.078825	0.375263	0.009181	0.011746	0.047787	0.754646	0.014402	1.53523	0.011833
2.81813	72.7641	2.26921	2.70627	5.6448	85.9957	2.79533	51.5962	0.495522
5.91744	37.0257	2.00217	2.74663	4.3937	42.6625	4.39747	59.4336	2.07784
0.011031	0.142927	0.011913	0.003613	0.03934	0.108427	0.013917	0.261478	0.008844
2.1312	61.1593	0.479226	0.928642	0.819168	12.2408	0.930145	17.8618	0.366644
0.376971	3.08703	0.72064	0.082178	3.25305	3.43837	1.67584	7.94935	0.215863
10.6527	189.724	2.10236	4.75689	2.67382	37.6939	1.97705	77.8094	1.63256
11.1462	199.037	2.15862	4.99612	2.82482	38.9719	2.08599	79.0244	1.71099
7.5192	121.93	1.85446	3.36204	5.2075	46.5587	2.4473	64.2996	1.50496
0.20808	1.61794	0.125763	0.113393	0.182473	1.60223	0.087172	3.32631	0.080384
0.012042	0.120703	0.001531	0.004032	0.00217	0.016795	0.002443	0.067905	0.009642
142.497	1498.05	87.8299	87.5402	83.4715	896.855	89.5997	790.73	46.1295
1.28295	23.3772	0.360103	0.547354	0.638906	10.1191	0.461782	18.6532	0.336553
0.000162	0.006011	0.000257	0.000125	0.001627	0.021265	0.000583	0.001689	0.00013
0.065073	0.414746	0.046132	0.029919	0.047541	0.235119	0.060805	0.261294	0.020909
1	1	1	1	1	1	1	1	1

0.031871	0.173953	0.053576	0.010855	0.036968	0.159411	0.026391	0.121658	0.010373
23.6465	207.013	17.2073	9.35419	16.8156	119.273	15.0254	81.9846	7.38723
1.56127	75.7865	0.520746	0.995602	0.806564	8.89584	0.808799	8.23423	0.452826
0.048305	0.440822	0.012394	0.018051	0.016041	0.184452	0.014007	0.6076	0.009276
0.032683	0.219544	0.01187	0.012239	0.028658	0.220531	0.016739	0.549675	0.015001

CHL-3	CHL-4	CHL-5	CHL-6	CHL-7	CHL-8	CHL-9	CHL-10	CHL-11
0.006386	0.045195	0.000674	0.012539	0.000173	0.008182	0.00837	0.004848	0.009094
0.688186	0.792481	0.098404	0.775184	0.051307	0.155713	0.512725	0.178538	0.816424
0.237585	0.276275	0.021937	0.209587	0.008443	0.092761	0.052064	0.069753	0.116928
0.949963	1.35211	0.104	1.10727	0.050499	0.140934	0.150659	0.143475	0.700303
1.19618	1.61733	0.173265	0.393011	0.150178	0.279799	0.200362	0.381845	0.728827
0.873529	0.290692	0.190233	0.226248	0.210154	0.096697	0.452876	0.173802	0.428093
0.135546	0.022571	0.067695	0.052698	0.078265	0.011554	0.906229	0.013504	0.115448
0.449881	1.59045	0.025774	0.226686	0.034358	0.019916	0.05343	0.139165	0.120805
0.478986	0.167572	0.116934	0.266127	0.064303	0.000209	0.419895	0.000996	0.138191
3.408	5.04518	1.11228	2.60053	1.53367	1.02066	2.55048	0.68492	5.11821
0.615904	1.57681	0.168781	0.457029	0.182183	0.155556	0.434231	0.213521	0.561729
0.000479	0.002946	0.000552	0.001825	0.000431	0.001587	0.00136	0.001616	0.001326
3.16974	3.89781	0.053462	0.222404	0.091194	0.025731	0.099251	0.03471	0.987067
0.042994	0.261499	0.007293	0.01788	0.006717	0.009099	0.018689	0.016566	0.018496
1.29468	9.01411	0.308317	0.901477	0.11984	0.160533	1.05141	0.221327	1.91443
0.212107	0.044059	0.010588	0.088811	0.043157	0.000283	0.090992	0.0021	0.033083
0.069967	0.278356	0.019583	0.178882	0.012782	0.049446	0.044309	0.058196	0.055526
8.8651	4.77585	0.802654	16.1537	0.521745	0.02647	3.24482	0.03824	5.27955
64.2461	140.566	3.05154	63.7206	2.47395	3.70886	3.45417	4.48386	55.1934
1.30302	4.61159	0.135495	1.13234	0.040356	0.106337	0.175396	0.111679	1.09895
0.848568	2.55177	0.012897	0	0.027538	0.016249	0.039811	0.024428	0.03507
5.14515	26.9435	0.158704	6.60692	0.197351	0.145055	0.446272	0.18762	4.20456
8.13357	37.0184	0.222057	8.63111	0.088549	0.018799	0.900177	0.040585	7.0416
204.988	28.8416	15.0278	313.178	15.7454	45.8289	48.6279	49.8279	391.999
0.173065	0.180122	0.027338	0.045504	0	0.01344	0	0.009083	0
73.6248	342.752	5.52165	121.703	4.57091	7.06704	9.98467	10.002	132.659
6.65598	35.5358	0.121947	6.19949	0.397985	1.82955	0.438838	1.54082	16.7698
227.847	379.838	16.1676	142.262	14.1211	19.0979	40.0571	22.6729	262.226
6.40822	5.97637	0.136358	7.9608	0.072547	0.004125	0.629673	0.007868	7.00575
10.0163	17.9087	1.88608	7.24355	3.02989	1.40489	2.65324	2.41779	115.504
0.979095	7.77491	0.013878	0.868582	0.035365	0.081089	0.037867	0.134403	1.20702
1.27349	13.6621	0.220606	1.72461	0.044602	1.03964	0.869538	0.065313	10.7821
0.159524	0.160556	0.002747	0.200297	0.000848	0.001944	0.009437	0.002141	0.120598
4.42031	6.11301	0.069545	3.67137	0.08737	0.162709	0.224988	0.21888	2.58335
41.335	113.361	1.43863	71.4444	1.42982	1.26028	3.7558	0.806508	86.0044
11.7792	23.0658	0.52759	13.3355	0.480299	0.327081	1.38481	0.289202	24.9573
0.562551	2.06158	0.010475	0.773547	0.003646	0	0.022259	3.11E-05	0.896225
49.312	39.9938	1.55041	58.7026	1.76836	0.292173	1.9294	0.451916	20.0017
54.5423	75.3175	0.884472	46.712	1.15342	2.08399	2.81039	2.78391	32.7151
0.201216	0.162972	0.003857	0.10897	0.006597	0.009365	0.019678	0.009454	0.098702
13.9116	118.127	0.243032	11.861	0.516837	0.080489	0.800937	0.084728	16.0149
4.26575	5.87079	0.076716	3.43383	0.120563	0.371649	0.955853	0.711555	3.57935
42.4525	283.779	0.826726	38.2099	2.50216	5.3703	1.93062	5.56628	119.481
44.3863	297.665	0.879839	39.4756	2.63403	5.53877	2.14254	5.75757	125.845
49.9266	306.325	0.871234	40.7028	1.59037	3.87687	2.4268	5.30297	55.3264
1.11281	2.84903	0.072901	1.60501	0.054301	0.036765	0.167702	0.023941	1.35239
0.037031	0.439782	9.63E-05	0.005341	0.001602	0.001768	0.001176	0.006419	0.025414
919.326	1464.6	50.8132	1155.04	38.798	96.5584	86.5947	87.9711	996.111
9.95813	78.3873	0.159469	8.36018	0.211645	0.6565	0.492804	0.630782	11.8583
0.003012	0.002514	0.000102	0.004293	8.16E-05	0.000985	0.000331	0.000264	0.003288
0.495692	0.414246	0.03367	0.453758	0.014352	0.092556	0.0809	0.092424	0.2761
1	1	1	1	1	1	1	1	1

0.120596	0.180921	0.020384	0.181296	0.009508	0.028211	0.02616	0.027091	0.088714
170.424	150.017	8.21832	192.866	5.25644	0.476746	18.8496	0.227559	115.668
12.2287	74.9725	0.779453	11.2707	0.677658	0.811761	0.565344	2.05127	7.36082
0.020963	1.57046	0.000135	0.639427	0.009614	0.09178	0.020929	0.075114	0.234292
0.311866	0.320098	0.005302	0.346295	0.008157	0.000642	0.015539	0.001401	0.331851

CHL-12	CHL-13	CHL-14	CHL-15
0	0.001209	0.007888	0.002247
1.27846	0.175355	0.189067	0.769989
0.104007	0.016513	0.100563	0.075197
1.02319	0.074808	0.146505	0.433805
1.07351	0.082033	0.146463	0.120769
1.01923	0.178573	0.098301	0.697817
0.496943	0.036638	0.013288	0.156877
0.20318	0.014067	0.042646	0.200381
0.194898	0.227028	0.0006	0.05893
4.68937	1.78705	0.435639	1.50424
1.00258	0.118393	0.136767	0.320196
0	0.000362	0.002591	0.001063
7.47293	0.156881	0.021514	1.02807
0.045062	0.00216	0.010896	0.008222
2.31793	0.105539	0.254783	0.612908
0.16993	0.012456	0.002659	0.03407
0.383518	0.011466	0.084181	0.036925
12.9958	0.786636	0.048227	3.23028
113.345	5.8134	2.73426	51.6485
3.1634	0.090714	0.158907	0.42154
1.6057	0.010916	0.02158	0.2804
9.61059	0.382888	0.200647	1.18336
18.9444	0.384672	0.021594	2.23584
450.235	28.1333	43.9792	212.183
0.162303	0	0.012486	0.256268
83.6738	9.56674	10.7305	59.6094
9.18637	0.407363	1.07548	2.43865
337.733	30.2716	28.7486	172.272
14.9729	0.303645	0.015065	1.59056
82.0851	2.20531	1.23541	18.4584
1.45209	0.048993	0.073512	0.440981
5.09403	0.098089	0.882399	0.721614
0.287307	0.00702	0.003686	0.075523
8.38526	0.208819	0.18438	2.44048
66.3287	3.88819	1.01917	30.4084
34.0322	1.43797	0.511864	10.5834
1.14058	0.023255	0.000268	0.117226
84.4586	3.70913	0.447973	34.5801
107.388	2.65272	2.3318	30.5896
0.208004	0.005625	0.028439	0.071398
22.6052	0.739253	0.204032	4.9915
5.29719	0.156857	0.803339	2.02157
76.7078	3.52925	3.55773	20.3563
79.9868	3.68777	3.33787	21.0318
74.0755	2.6899	4.6403	19.5083
3.70136	0.09329	0.023399	0.628554
0.048543	0.001233	0.003389	0.006382
1566.75	65.9616	103.906	523.327
10.5371	0.389483	0.820324	3.60129
0.002973	0.000231	0.00089	0.001417
0.443322	0.024893	0.114349	0.220742
1	1	1	1

0.256912	0.011045	0.029981	0.08971
242.633	9.25112	0.842567	71.6406
12.0257	0.710435	0.921324	8.01811
0.316478	0.017625	0.053919	0.173866
0.517282	0.017617	0.00072	0.113009
